# Gene regulatory network inference and analysis of multidrug-resistant *Pseudomonas aeruginosa*

**DOI:** 10.1590/0074-02760190105

**Published:** 2019-08-05

**Authors:** Fernando Medeiros, Ana Paula Barbosa do Nascimento, Marcelo Trindade dos Santos, Ana Paula D’Alincourt Carvalho-Assef, Fabricio Alves Barbosa da Silva

**Affiliations:** 1 Fundação Oswaldo Cruz, Programa de Computação Científica, Rio de Janeiro, RJ, Brasil; 2 Laboratório Nacional de Computação Científica, Petrópolis, RJ, Brasil; 3 Fundação Oswaldo Cruz, Instituto Oswaldo Cruz, Laboratório de Pesquisa em Infecção Hospitalar, Rio de Janeiro, RJ, Brasil

**Keywords:** Pseudomonas aeruginosa, gene regulatory network, multidrug resistance

## Abstract

**BACKGROUND:**

Healthcare-associated infections caused by bacteria such as *Pseudomonas aeruginosa* are a major public health problem worldwide. Gene regulatory networks (GRN) computationally represent interactions among regulatory genes and their targets. They are an important approach to help understand bacterial behaviour and to provide novel ways of overcoming scientific challenges, including the identification of potential therapeutic targets and the development of new drugs.

**OBJECTIVES:**

The goal of this study was to reconstruct the multidrug-resistant (MDR) *P. aeruginosa* GRN and to analyse its topological properties.

**METHODS:**

The methodology used in this study was based on gene orthology inference using the reciprocal best hit method. We used the genome of *P. aeruginosa* CCBH4851 as the basis of the reconstruction process. This MDR strain is representative of the sequence type 277, which was involved in an endemic outbreak in Brazil.

**FINDINGS:**

We obtained a network with a larger number of regulatory genes, target genes and interactions as compared to the previously reported network. Topological analysis results are in accordance with the complex network representation of biological processes.

**MAIN CONCLUSIONS:**

The properties of the network were consistent with the biological features of *P. aeruginosa*. To the best of our knowledge, the *P. aeruginosa* GRN presented here is the most complete version available to date.

Healthcare-associated infections (HAI) are a major global public health problem as they increase the morbidity and mortality rates of hospitalised individuals. HAI are often caused by multidrug-resistant (MDR) bacteria, such as *Pseudomonas aeruginosa*, especially in immunocompromised patients. In Brazil, *P. aeruginosa* was ranked as the fifth most common causative agent of HAI in patients hospitalised in adult and paediatric intensive care units, and nearly 35% of the reported strains are resistant to carbapenems, a class of antibiotics widely used to treat *P. aeruginosa* infections.^1^ In fact, individuals infected with MDR *P. aeruginosa* clones have a higher mortality rate (44.6%) compared to those infected with non-MDR clones (24.8%).^2^

*P. aeruginosa* is a versatile pathogen that can cause several types of infections affecting the lower respiratory tract, skin, urinary tract and eyes, leading to bacteraemia, endocarditis and other complications. *P. aeruginosa* infections are difficult to treat as the therapeutic options are becoming increasingly limited. Biofilm formation and the presence of intrinsic resistance-associated genes are examples of the mechanisms that *P. aeruginosa* employs to resist chemotherapy. In addition, this bacterium can become resistant to a broad range of antibiotics through the acquisition of new resistance mechanisms via horizontal gene transfer.^3^ ^-^ ^5^

In 2000, the genome sequence of *P. aeruginosa* PAO1 strain was published, providing data regarding its genetic complexity and ecological versatility.^6^ The PAO1 strain is sensitive to most clinically used antimicrobial agents and has been extensively studied since the publication of its genomic sequence.

In 2003, the first clinical isolate of an MDR *P. aeruginosa* strain carrying the carbapenemase gene, *bla*_SPM-1_, was identified in Brazil. The SPM-1 protein is a metallo-β-lactamase that confers resistance to almost all classes of beta-lactams.^7^ Most of the SPM-producing isolates belong to sequence type (ST) 277, as indicated by the multilocus sequence typing (MLST). The ST277 has been associated with hospital outbreaks in several Brazilian states and has been isolated from hospital sewage systems and rivers.^8^ ^-^ ^10^

In recent years, researchers have applied mathematical methods to generate computational models to study the behaviour of certain organisms. This approach has contributed to the development of new products, the improvement and acceleration of existing health policies and research into novel ways of overcoming scientific challenges. It is often based on the construction of biological networks and the analysis of gene regulatory, metabolic and signal transduction pathways and/or protein-protein interactions.^11^

A gene regulatory network (GRN) is a collection of transcription factors (TFs) that interact with each other and with other molecules in the cell to regulate mRNA and protein expression levels. In 2011, Galán-Vásquez et al.^12^ published the first *P. aeruginosa* GRN and ana-lysed its main topological properties and interactions between its regulatory components.

In the present study, we describe the reconstruction of the GRN of an MDR *P. aeruginosa* strain, including all currently available curated biological data. This reconstruction was based on *P. aeruginosa* CCBH4851, a strain representative of the ST277, which was involved in an endemic outbreak in Brazil. This strain shows resistance to all antimicrobials of clinical importance with the exception of polymyxin B, has several mechanisms of resistance and mobile genetic elements.^13^ The implications of the choice of an MDR strain as the basis of the GRN reconstruction are discussed in this study. In addition, the topological properties of GRN were analysed, and regulators, target genes (TGs), TFs, auto-activation mechanisms, influential genes and network motifs were characterised.

## MATERIALS AND METHODS

*Bacterial strains* - In this study, we reconstructed a GRN for *P. aeruginosa* CCBH4851. This strain is deposited at the Culture Collection of Hospital-Acquired Bacteria (CCBH) located at the Laboratório de Pesquisa em Infecção Hospitalar, Instituto Oswaldo Cruz/Fundação Oswaldo Cruz (Fiocruz) (WDCM947; 39 CGEN022/2010) and its genome sequence is available in the GenBank database (accession CP021380).^13^ For the orthology analysis, *P. aeruginosa* PAO1,^6^ *P. aeruginosa* PA7^14^ and *P. aeruginosa* UCBPP-PA14 (PA14)^15^ were chosen as reference strains.

**Fig. 1:**
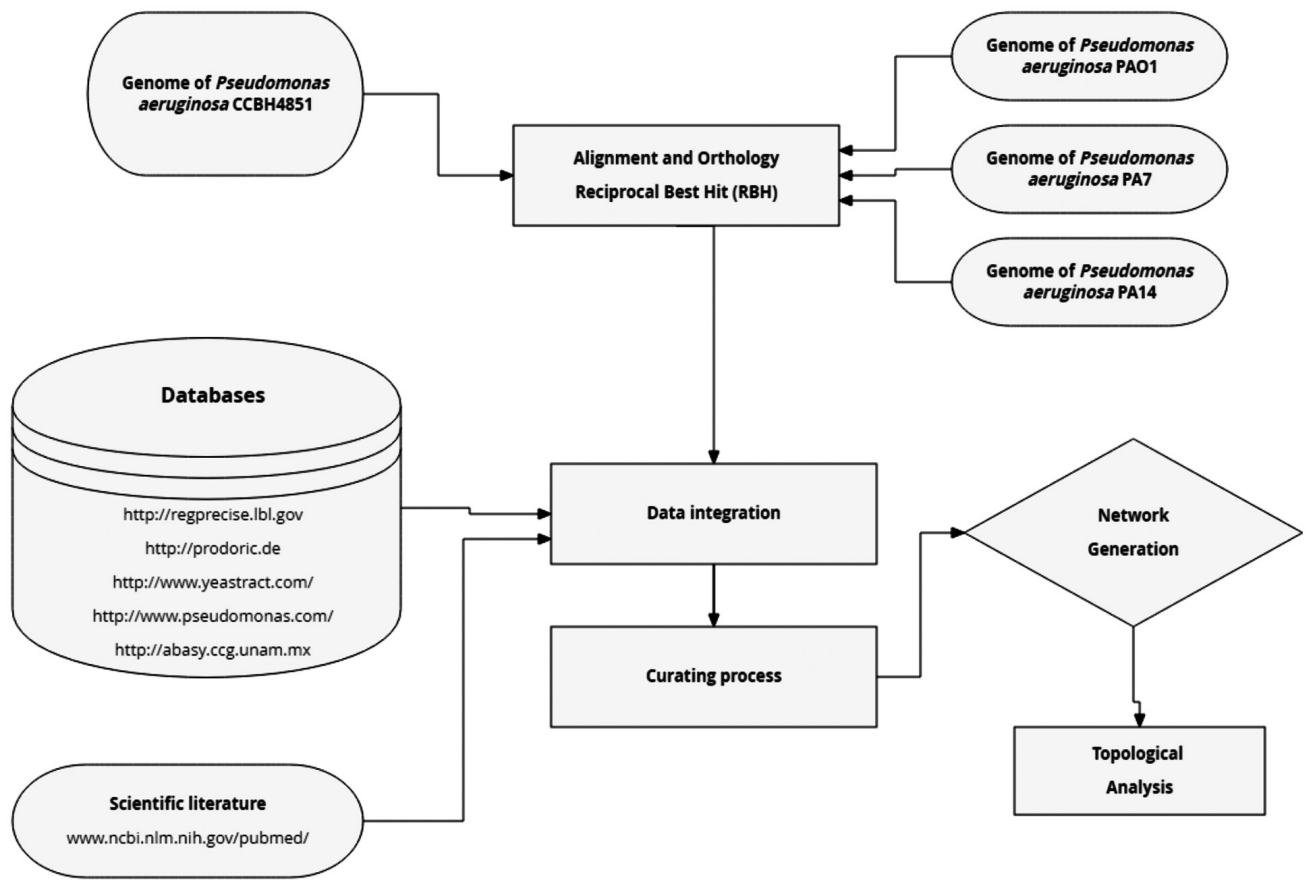
overview of the general strategy for reconstruction of the *Pseudomonas aeruginosa* gene regulatory network (GRN). The process began with the alignment of the *P. aeruginosa* CCBH4851 genome and the three reference strains. Next, the reciprocal best hits (RBH) method was applied and the resulting genes were compared against the gene regulatory databases listed in the “Databases” box. Data obtained from these databases were then integrated and submitted to the curation process, which aims to solve network inconsistencies. Finally, the GRN was generated and its topology was analysed.

*Orthology-based model generation* - Fitch^16^ defines orthologs as genes that diverged after a speciation event, but share a common ancestor. The most common approach to find orthologs is the reciprocal best hits (RBH) method.^17^ The regulatory interactions between TFs and TGs in *P. aeruginosa* PAO1, *P. aeruginosa* PA14 and *P. aeruginosa* PA7 strains were propagated to the reconstructed *P. aeruginosa* CCBH4851 network if both the TF and the TG formed RBH. The criterion used to define an orthologous relationship was the existence of RBH between the two genomes. Two genes, x and x’, of the genomes X and X’, respectively, were considered orthologs if they were also RBH, i.e., if upon aligning the sequence of x against the gene list of X’, we obtained x’ as the best alignment, and if upon aligning the sequence of x’ against the gene list of X, we obtained x as the best hit. Once the complete set of RBHs between X and X’ genomes was obtained, a regulatory interaction between a TF (gene x) and a TG (gene y) was propagated from the reference network to CCBH4851, if both the TF and the TG had their respective RBH in the CCBH4851 genome. The propagation of a regulatory interaction, x-y, from the reference genome X, held if the pair, x’-y’, existed in the genome X’, such that both (x, x’) and (y, y’) were RBH pairs. One disadvantage of the RBH method is its inability to detect multi-to-multi orthologous relationships. In this case, RBH only picks the hit with the best score alignment, resulting in false negatives. To prevent these false negatives, when a gene presented no ortholog in genome X’, manual curation was performed as follows: the protein sequence encoded by gene x of genome X was searched against genome X’ using the BLASTX algorithm. If the search returned two or more hits, the neighbouring region of each hit was assessed to determine which gene in genome X’ was orthologous to that specific protein, matching its genomic context. If the search returned no hits, the gene was considered to have no ortholog in genome X’. This test for the propagation of regulatory interactions was performed with all known interactions in PAO1, PA7 and PA14. All-against-all alignments were performed using the BLASTP program with the following stringent parameters: identity ≥ 90%, coverage ≥ 90% and E value cut-off of 1 e-5. Fig. 1 presents an overview of the reconstruction process.

*Identification of RBH* - An algorithm was implemented using the Python programming language to automate and generate a list of RBH in a tabular format. Regulators and TGs were then identified and separated into a single table, thus extending the work done by Galán-Vásquez et al.^12^

*Data integration* - The data integration process brought together biological information from all strains with the aim of organising biological knowledge. The final network table is available as Supplementary data. This table is organised into six columns: Regulatory gene, Ortholog of the regulatory gene, Target gene, Ortholog of the target gene, Mode of regulation and Reference. The first column lists the regulatory genes of *P. aeruginosa* CCBH4851, the second column contains orthologs of regulatory genes in the reference strain (PAO1, PA7 or PA14), the third column refers to the target gene in CCBH4851, the fourth column lists orthologs of TGs in the reference strain, the fifth column describes the mode of regulation and the sixth column indicates the source of the corresponding data.

*Curation process* - Our group has developed a web application to support the curation of biological networks. This web application, called CurSystem^18^ (available from: http://Pseudomonas.procc.fiocruz.br:8185/CurSystem) provides support for distributed, asynchronous interaction among specialists in the fields of biochemistry, chemistry, microbiology, molecular biology, computational and systems biology, computer and systems engineering and physics. This tool was used to select specific gene interactions, discuss their main peculiarities and determine whether they would be part of the network. This stage was fundamental to excluding doubtful biological information from the network.

*Network generation and computational analysis* - The R programming language and the open-source software, RStudio were used for network generation and computational analysis.^19^ Analysis of degree, centrality, clustering coefficient, connectivity, cycles, paths and hierarchical levels was performed according to previously described methods.^12^ ^,^ ^20^ We used the dplyr, tibble, readr, igraph and scales packages for these analyses. The igraph package was used for the computation of feed-forward loop (FFL) motifs (function triad_census). Network degree-entropy was measured according to the method described by Breitkreutz et al.^21^

All data and code are available as Supplementary data.

## RESULTS

*General features of the* GRN - The *P. aeruginosa* network reconstruction resulted in a total of 1,046 genes, of which 42 behaved as regulatory genes, 96 as both regulatory and TGs (i.e., a TF was influenced by another TF in the network) and 908 were TGs. We identified 1,576 regulatory interactions between regulators and their TGs. Altogether, these genes represented approximately 16.52% of the genome of *P. aeruginosa* CCBH4851, which was used as the model organism in this study. Despite the apparent low coverage, we included most of the TFs with known functions among the 138 regulators in the *P. aeruginosa* CCBH4851 network. The number of regulatory genes, TGs, and interactions represented an increase of 44.92, 34.69 and 35.27%, respectively, compared to previous study.^12^ Network enrichment was not the only result observed in the *P. aeruginosa* CCBH4851 GRN reconstruction. As the reconstruction was based on the RBH method that involved comparing the CCBH4851 genome annotation with reference strains, it was not possible to infer an orthologous relationship for some genes, particularly *oprD* and *mexZ*, which are genes involved in antibiotic resistance mechanisms. The curation process revealed that these genes were either fully absent or annotated as pseudogenes in CCBH4851. A pseudogene is a DNA sequence that resembles a gene in the reference genome, but has modifications such as point mutations, insertions, deletions, premature stop codons or frameshifts, making it impossible to determine if its product is still functional in the target organism, without further experimentation. The lack of orthology resulted in the exclusion of these genes from the *P. aeruginosa* CCBH4851 GRN. In addition, certain regulatory genes and its interactions were kept as described in the previous network,^12^ and in the databases and/or scientific literature used. For example, *ihf* (integration host factor) represents not a single gene, but a complex composed of the products of the *himA* and *himD* genes that act in combination as a TF for several TGs. However, regulatory systems such as quorum sensing or two-component systems are often formed by a pair of genes, but only one of them is able to bind to the promoter region of the target gene. However, both genes were listed as regulatory genes. Therefore, we were able to maintain equivalent notations to previous network.^12^

**Fig. 2:**
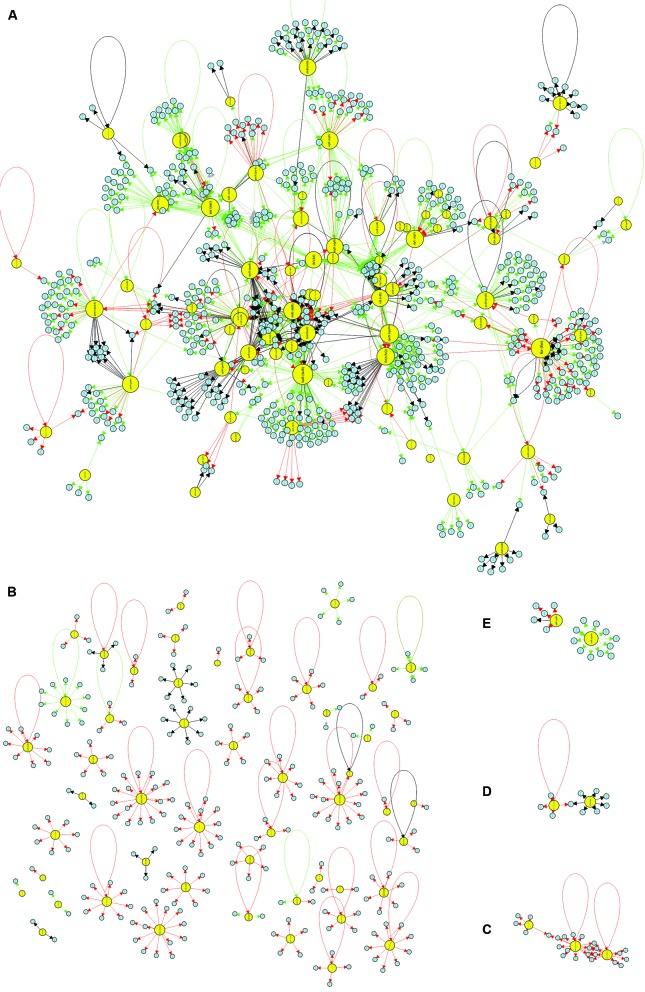
visualisation of the *Pseudomonas aeruginosa* CCBH4851 gene regulatory network (GRN). Yellow circles indicate regulatory genes, light blue circles indicate target genes (TGs), black lines indicate an unknown mode of regulation, green lines indicate activation, red lines indicate repression and grey lines indicate a dual mode of regulation. A: the GRN large highly connected network component; B: all regulatory and TGs that have no connections with the component depicted in A; C-E: clusters of lower connectivity compared to the component depicted in A. All figures are presented with higher resolution in the Supplementary data.

*Basic network topological analysis: number of vertices, number of edges and density* - We identified 1,576 edges in the CCBH4851 network. These interactions were classified into four types: activation (“+”), repression (“-”), dual (“d”, when the regulatory gene could act as an activator or a repressor, depending on the conditions), and unknown (“?”). An illustration of the CCBH4851 GRN is presented in Fig. 2. Network density is a measure of the interconnectivity between vertices. It is the ratio of the actual number of edges in the network and the maximum possible number of edges. The regulatory network of the CCBH4851 strain had a density of 1.44 e-03, which was slightly lower than the observed density of 2.12 e-03 for the PAO1 strain, but was of the same order of magnitude. A network diameter indicates the path length between the two most distant nodes. The CCBH4851 GRN had a diameter of 12 nodes, while the previous network had a diameter of 9 nodes. Another measure, the average path distance, also known as the average shortest path, is the average distance between two nodes. While the previous network showed an average path distance of 4.08, the CCBH5851 GRN had an average path distance of 4.80.^12^ ^,^ ^22^

**Fig. 3:**
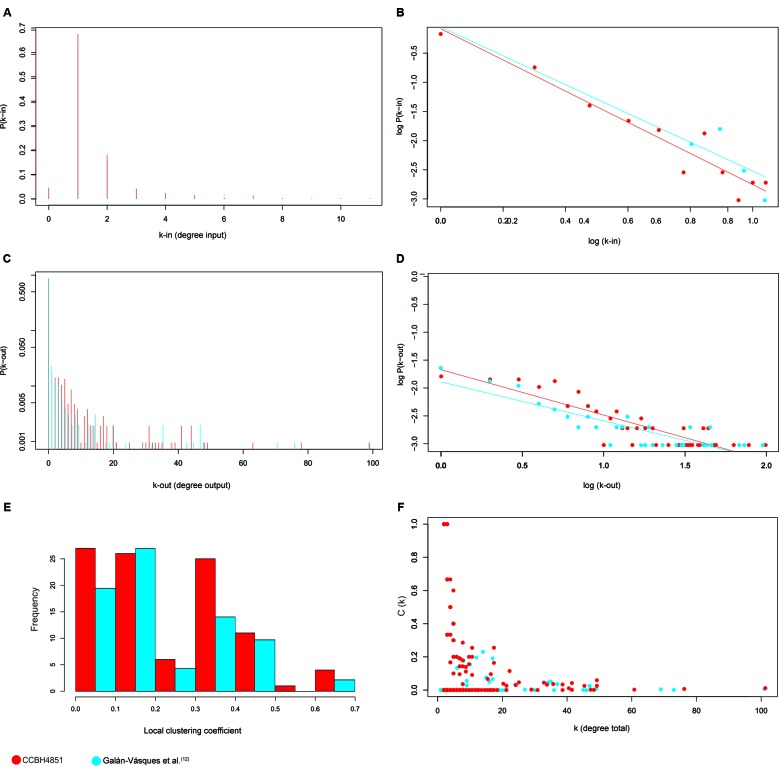
graphical representation of topological measurements of the *Pseudomonas aeruginosa* CCBH4851 gene regulatory network (GRN) (red) compared to the previously published network^12^ (blue). A, B: incoming degree distribution of the *P. aeruginosa* CCBH4851 GRN; C, D: outgoing distribution of the *P. aeruginosa* CCBH4851 GRN. For clarity, the distributions are plotted both on a linear (A, C) and on a logarithmic scale (B, D); E: local clustering coefficient distribution; F: clustering coefficient by degree.

The degree *k(i)* of a vertex *i* is defined as its number of edges. Edges in directed networks can be of two types: they can “depart” from or “arrive” at node *i*, thus defining them as “incoming” (*k-in*) or “outgoing” (*k-out*) degrees, respectively. In the CCBH4851 GRN, each vertex was, on average, connected to three other vertices, which was the same as reported for the PAO1 GRN. Fig. 3 illustrates the incoming (3A, B) and outgoing (3C, D) degree distributions for the CCBH4851 GRN.

Scale-free is a common topology classification associated with biological networks, corresponding to complex networks in which the degree distribution follows a power law. In scale-free networks, most nodes (vertices) have few connections and few nodes have a large number of connections. Therefore, scale-free networks are dominated by a relatively small number of high-degree nodes, generally referred to as hubs.^23^

The degree distribution can be approximated by:

$$P\left(k\right)\sim {Ak}^{-y}\;(1)$$

Equation 1 corresponds to a power-law distribution and the exponent, γ, is its degree exponent.^24^ The degree distribution shown in Fig. 3B, D is on a double logarithmic axis and the straight line is consistent with a power-law distribution. For *k-in*, the estimated value for γ was 2.89, which is very close to the value reported for the reference network (γ =2.717).^12^

*Clustering coefficient distribution* - Given a node *i* with *m(i)* neighbours in a directed network, the maximum number of edges connecting the elements of this neighbourhood is given by *m*_*max*_ *(i) = m(i)(m(i)-1)*. The local clustering coefficient, *C(i)*, is defined as the ratio of the actual number of edges *N(i)* occurring in node *i* neighbourhood and *m*_*max*_ *(i).* ^25^ The local clustering coefficient is defined as *C(i) = N(i)/m*_*max*_ *(i)*. In GRNs, the local clustering coefficient *C(i)* is interpreted as the interaction between genes forming the regulatory groups. The distribution of local clustering coefficients can be seen in Fig. 3E. 

The global clustering coefficient is proportional to the number of triangles present in the network, disregarding the directionality of the edges. A tringle is a set of three nodes with at least two connections between them. Triangles can be closed, with three connections within the set, or open triangles, with only two edges. The global clustering coefficient, *C*, is the ratio between the number of closed triangles and the total number of triangles (closed or open) in the network. The CCBH4851 network had a global clustering coefficient equal to 3.2 e-02.

Another interesting feature to observe is the correlation between the local clustering coefficient *C(i)* and the degree *k(i)*, as shown in the scatter plot in Fig. 3F. The observed correlation was negative and Fig. 3 also shows that the vertices with high degree, *k*, corresponded to the same vertices with null clustering coefficients, while the vertices that formed clusters had low degrees. This observation confirmed that strongly cohesive groups are exceptions in the network and are formed by a small number of genes. These results were obtained for both the CCBH4851 GRN and the previously published *P. aeruginosa* GRN.^12^

*Connectivity* - Network connectivity is a concept that reflects the associations between every pair of genes. Nodes were considered a part of a connected component when they interacted through a direct or an indirect link (intermediate connections). In the connectivity analysis, network interactions were considered undirected. Similar to the reference GRN, the CCBH4851 network was disconnected. It showed one large connected component (including 751 nodes) and more than 48 small connected components, which is larger than the previously reported network.^12^ However, there may be several reasons for the network being disconnected at specific points. Firstly, this may represent the natural behaviour of the organism, i.e., not all genes in a complex genome are linked, because cellular processes can be compartmentalised or global, constitutive or growth phase-dependent. Secondly, there may have been insufficient biological information to infer the interactions. Thirdly, *P. aeruginosa* genomes generally maintain a conserved core component, which accounts for the majority of the genome. However, additional strain-specific blocks of genes are acquired by horizontal gene transfer, as a result of evolutionary events, and these can result in a decreased degree of similarity with reference strains and an increased degree of similarity with newly reported strains. This process can result in the loss of existing interactions or a gain of interactions still not fully described, thus lacking a connection with other components in the network.

*Dominant activity and autoregulation* - The analysis of the frequency of the different modes of regulation indicated that activation is the predominant type of regulation mode in the CCBH4851 network, with frequency values very similar to those previously observed for the *P. aeruginosa* GRN. Overall, 48.92% of the interactions were of the activation mode, 28.8% were of the repression mode and 22.27% were of dual or unknown modes. Although the distribution pattern was maintained, a significant enrichment was observed in negative and unknown regulation modes. When considering autoregulation, i.e., a gene regulating its own expression, the CCBH5851 GRN showed a predominance of negative autoregulatory motifs, which differs from the findings of Galán-Vásquez et al.^12^

*Motifs* - The existence of cycles or motifs in biological networks is a necessary condition for the existence of multiple stationary states or attractors. In GRNs, the most common 3-genes motif is the FFL. The FFL motif comprises a gene A that regulates gene B. Then, both A and B regulate gene C. There are two types of FFL motifs: (i) coherent, where the regulatory effect of both paths, direct and indirect, are the same and (ii) incoherent, where the regulatory effects are different. In this study, we computed the total number of FFL motifs: the number of coherent type I FFL motifs (where all interactions are activations) and the number of incoherent type II motifs, (where all interactions are repressions).^26^ The CCBH4851 GRN had a larger number of FFL motifs (when considering all variations), when compared to the GRN published by Galán-Vásquez et al. The coherent type I FFL motif was the most abundant in both networks, with 82 representatives in the PAO1 GRN and 79 in the CCBH4851 GRN. Meanwhile, there were four incoherent type II FFL motifs in the CCBH4851 GRN compared with three in the previously described network.^12^

**Fig. 4:**
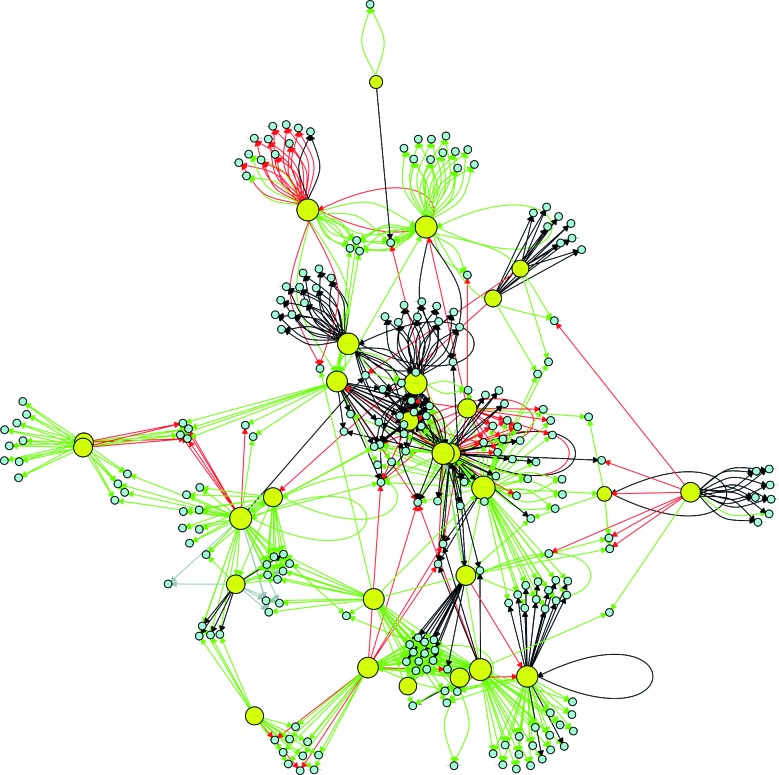
connectivity relationships among the 30 most influential hubs of the *Pseudomonas aeruginosa* CCBH4851 gene regulatory network. Yellow circles indicate regulatory genes considered hubs, light blue circles indicate target genes, black lines indicate an unknown mode of regulation, green lines indicate activation, red lines indicate repression and grey lines indicate a dual mode of regulation.

*Hubs* - Identifying the most influential genes in a gene transcription network is a key step in determining therapeutic targets against an infectious agent. One way to identify possible targets is to identify so-called network hubs. Different definitions of a hub can be applied in the context of complex network theory. One method of identifying a hub is to determine which vertices have the highest *k-out* degrees in order to identify, in the case of a GRN, the genes with the greatest influence on target regulation. According to Vandereyken et al.,^27^ the exact number of interactions that characterise a hub, also called the degree threshold, differs among different studies. Some studies have shown that the minimum number is five, whereas others have reported eight, 10, 20 or even 50 as the minimum number. In the present study, the degree threshold was defined as the average of the number of connections of all nodes having at least two edges. The application of this definition resulted in a cut-off value of 16 connections. Table I shows the 30 most influential hubs in the *P. aeruginosa* GRN. After pinpointing the hubs, an analysis was performed to determine whether they are interconnected (through direct or indirect interactions). Only two hubs were found to not be interconnected, *np20* and PA4851_19380 (homologous to PA1520). The remaining hubs had a direct (when a hub affects the regulation of another hub) or indirect (when hubs affect the regulation of the same group of TGs) connection to other hubs (Fig. 4). Node interactions that were not common among hubs were hidden to improve visualisation in Fig. 4.

**TABLE I:** The 30 most influential hubs of the *Pseudomonas aeruginosa* CCBH4851 GRN

Gene	Total number of connections (k-out)
*lasR*	99
*fur*	78
*rpoN*	63
*anr*	49
*mexT*	48
*rhlR*	44
*fleQ*	44
*algU*	41
*pmrA*	41
*argR*	39
*pvdS*	38
*rpoS*	35
*dnr*	34
*vfr*	33
*lasI*	32
*cbrB*	31
*algR*	31
*ihf*	30
*phoP*	29
*qscR*	25
*cysB*	21
*exsA*	20
*psrA*	20
*pprB*	18
*roxR*	18
*rsaL*	17
*roxS*	17
*np20*	17
*narL*	16
PA4851_19380	16

The summarised results comprising the network statistics are presented in Table II, which contains standard measures, such as the number of nodes, number of edges, number of autoregulatory motifs, diameter of the network and average path length. Other relevant measures were the number of coherent and incoherent feed-forward motifs, clustering coefficients and network entropy. Moreover, a comparison with data from the previous network^12^ is included in Table II.

**TABLE II:** Comparison of topological statistic measures between the gene regulatory networks (GRN) published by Galán-Vásquez et al.^(12)^ and the *Pseudomonas aeruginosa* CCBH4851 GRN

	Galán-Vásquez^(12)^ GRN	CCBH4851 GRN
Vertices	690	1046
Edges	1020	1576
Regulatory genes	76	138
Target genes	593	908
Positive regulation	779	772
Negative regulation	218	454
Dual regulation	11	13
Unknown regulation	12	337
Autoregulation (total)	29	72
Positive autoregulation	16	21
Negative autoregulation	13	39
Unknown autoregulation	-	12
Feed-forward loop motifs (total)^*a*^	137	218
Coherent type I feed-forward loop motifs^*a*^	82	79
Incoherent type II feed-forward loop motifs^*a*^	3	4
Density	2.12e-03	1.44e-03
Diameter	9	12
Average path length	4.08	4.80
Global clustering coefficient	2.28e-02	3.2e-02
Local clustering coefficient	2.5e-01	1.92e-01
Entropy	1.92	2.34

*a*: number of feed-forward loop motifs determined using the igraph package.

## DISCUSSION

The importance of gene regulation on metabolic, adaptive, pathogenic and antibiotic resistance capabilities is well known. The GRN reconstruction and analysis of a versatile pathogen, such as *P. aeruginosa,* particularly when based on an MDR strain, contributes to an increase in the knowledge of related cellular processes. Multidrug resistance can be conferred by a combination of factors that vary according to the antimicrobial class. For instance, carbapenem resistance in *P. aeruginosa* is manly conferred by mutations in *oprD* and/or by the presence of MBLs. Mutations or differential expression of efflux system genes are also contributing factors for both carbapenem and aminoglycoside resistance. Multidrug resistance can also occur through other mechanisms, including the acquisition of genes through horizontal transfer and punctual mutations, in multiple combinations.^28^ In addition, *P. aeruginosa* has the ability to form a biofilm, which can play a role in antibiotic penetration, antibiotic tolerance, the formation of persister cells and protection from the host immune system.^4^ Due to the limitations of graphical representation, it is difficult to incorporate data such as gene expression variation and point mutations or genes lacking experimental evidence in a GRN graph. Overall, we excluded *oprD*, *mexZ* and *pilA* genes from our network*.* As *oprD* is a target gene, its exclusion had a minor impact on the network topology. However, this gene is extremely important for the cell because *oprD* encodes an outer membrane porin, which is important for the absorption of carbapenems. The lack of OprD leads to low outer membrane permeability. However, the exclusion of *mexZ*, a regulatory gene, resulted in the exclusion of its node from the network, as well as the exclusion of the interactions with its TGs. The *mexZ* product represses the transcription of *mexX* and *mexY* genes. MexXY proteins are part of an efflux pump system, whose overexpression leads to aminoglycoside resistance through the extrusion of this family of compounds. However, MexXY overexpression needs to be experimentally established and cannot be represented in a graph. PilA is a major pilin protein associated with bacterial adherence through the type VI pilus machinery. Therefore, PilA has a great importance in pathogenesis. The *pilA* gene is also a target, which only shows regulatory influence upon itself. The advantageous effect of PilA loss to an MDR strain is unclear. One can hypothesise that this loss may be compensated by newly acquired genes, since CCBH4851 has a chromosome approximately 600 kb larger than PAO1. However, these alterations are common among MDR strains^29^ ^,^ ^30^ and constructing a network comprising these features may impact the dynamics of simulations designed to assess MDR bacteria behaviour based on the *P. aeruginosa* CCBH4851 GRN. Overall, the reconstruction reported here included additional regulators, TGs and new interactions described in the literature or included in curated databases since the last *P. aeruginosa* GRN publication.^12^ Several genes involved in virulence mechanisms were identified, such as those associated with the production of proteases and toxins, antimicrobial activity, iron uptake, antiphagocytosis, adherence and quorum sensing. In addition to the inclusion of new nodes and connections, previously identified nodes were excluded (by the curation process or the lack of homology) and interactions were revisited (due to genes whose regulatory effect had been recently elucidated). Two noteworthy examples of the included nodes and interactions are the regulatory effect of *fleQ* upon “*psl*” genes and the regulation of the efflux pump genes, *mexA*, *mexE*, and *oprH*, by *brlR*. The “*psl*” (polysaccharide synthesis locus) cluster comprises 15 exopolysaccharide biosynthesis-related genes organised in tandem. These genes are important for biofilm formation. The recently functionally characterised transcriptional regulator, BrlR, has biofilm-specific expression and plays a role in the antibiotic tolerance of biofilms by modulating the expression of efflux pump genes.^4^ Altogether, these alterations directly influenced the topological characteristics of the network. However, topology measures of the *P. aeruginosa* CCBH4851 GRN, such as node degree distribution and clustering coefficient, remained consistent with a scale-free network type. The degree distribution followed the power-law distribution (Fig. 3B, D), meaning that a small number of nodes had many connections and a large number of nodes had few connections. Moreover, the correlation between local clustering coefficient and node degree (Fig. 3F) showed that nodes with lower degrees had larger local clustering coefficients than nodes with higher degrees. Indeed, construction of several networks representing biological processes showed similar topological characteristics.^24^ ^,^ ^31^ As with other mathematical aspects of the network, topology *per se* was consistent with the type of network obtained, but it is important to ensure that these measures are consistent with biological observations. The reconstructed network showed a low-density value, which was compatible with the fact that networks representing natural phenomena often have low density, due to their structural and dynamic flexibility.^22^ The low density observed in the CCBH4851 GRN indicated that the nodes were not all interconnected. Biologically, in an organism such as *P. aeruginosa*, which has an average of 6,000 coding sequences, it is not expected that all genes maintain an interaction, since they are related to distinct biological processes that are not all dependent on each other and are triggered during different growth phases, thus corroborating the observed low density. In the same way, the global clustering coefficient and connectivity parameters are affected by these biological behaviours, resulting in the large number of connected components found in the CCBH4851 GRN. 

Although some nodes under positive regulation were lost (Table II), the most common regulatory activity found among CCBH4851 GRN interactions was activation. However, more than 50% of the autoregulation found was negative. This may be a consequence of the increase in negative regulation in the overall network interactions. A similar pattern is seen in the regulatory network of another member of the Gammaproteobacteria class, *Escherichia coli*, which exhibited the prevalence of negative autoregulation concurrently with the prevalence of positive regulation between TFs. The positive mode of regulation is important to ensure the continuity of biological processes. Adhesion, cell-to-cell signalling, production of virulence and resistance factors, biofilm formation, secretion of toxins and host-pathogen interaction factors are examples of processes that, once initiated, must reach a final stage in order to have the desired effect. In fact, we observed that genes such as *lasR*, *rlhR*, *pvdS*, *anr*, *dnr* and *algU*, involved in these types of processes mostly showed a positive mode of regulation in the CCBH4851 GRN. However, negative cycles are also important for life-sustaining cyclic processes, such as those involved in cellular homeostasis. This is the case for metabolic processes where genes such as *lexA*, *hutC, iscR*, *desT* and *mvat* (although involved in virulence factor biosynthesis, this gene regulates arginine metabolism), showed a negative mode of regulation as the predominant effect.^20^ Negative autoregulation is linked to cellular stability, providing a rapid response to variations in protein/toxin/metabolite concentrations, thus avoiding the energy cost of unneeded synthesis and avoiding undesired effects. Some examples of negative autoregulatory interactions included in the CCBH4851 network were *algZ*, *lexA*, *metR*, *ptxR* and *rsaL*. RsaL is a quorum-sensing repressor; LexA is involved in the SOS response; AlgZ is the transcriptional activator of AlgD, involved in alginate production; PtxR affects exotoxin A production; and MetR is involved in swarming motility and methionine synthesis. Overall, these autoregulatory genes tend to be further upstream in the regulatory chain. 

The dominance of the activation mode was also observed when analysing network motifs. Motifs are patterns of topological structures statistically overrepresented in the network. The number of FFL motifs, considering all variations, was 218 for the CCBH4851 GRN, but 137 for the GRN published by Galán-Vásquez et al.^12^ A common motif often related to transcriptional networks, the coherent FFL, was abundantly present in the CCBH4851 GRN (Table II).^26^ In particular, the coherent type I FFL motif, where all interactions are positive, was common in both GRNs. These motifs act as sign-sensitive delays, i.e., a circuit that responds rapidly to step-like stimuli in one direction (ON to OFF), and as a delay to steps in the opposite direction (OFF to ON). While the temporary removal of the stimulus ceases transcription, the activation of expression requires a persistent signal to carry on. Although less represented, the incoherent type II FFL motif was also found in the CCBH4851 GRN. In contrast to the coherent FFL, these motifs act as sign-sensitive accelerators, i.e., a circuit that responds rapidly to step-like stimuli in one direction, but not in the other direction.^26^ Overall, FFL motifs are important for the modulation of cellular processes according to environmental conditions.

One last characteristic revealed by the topological analysis was the presence of hubs. Hubs are nodes showing a large number of connections, a concept that is inherent to scale-free networks. As expected, analysis of the CCBH4851 GRN showed that genes known to have a great impact on the gene regulatory systems of *P. aeruginosa*, such as *lasR*, *fur*, *anr*, *mexT* and *algU*, were among the most influential hubs. These genes are involved in resistance, virulence, and pathogenicity mechanisms. LasR, for instance, directly activates the expression of 99 genes. The activity of LasR depends on the presence and binding of *N*-3-oxo-dodecanoyl-L-homoserine lactone (C12). Once bound, the LasR-C12 complex coordinates the expression of TGs, including many genes encoding factors involved in virulence and cell density.^32^ In addition, *fur* is a global regulator of iron uptake, *rpoN* is an alternative sigma factor, *mexT* regulates an efflux pump system and several virulence factors and *anr* is responsible for the regulation of anaerobic adaptation processes. All of these genes are known to control the expression of many other genes. We observed that, even though a few hubs remained unconnected, most of the influential genes belonged to the major connected component. This interaction can be direct, as in case of the positive effect of LasR on *rlhR* transcription, or indirect, when hubs are regulating the same targets, i.e*.*, involved in the regulation of the same processes, such as *fur* and *algU*, both of which affect the expression of *phuR*, which encodes a member of a heme uptake system that facilitates host iron acquisition.^33^ ^,^ ^34^ Another example is the regulation of “*alg*” genes by *algU*, *rpoN* and *cysB*, which are not directly connected, but are related through their effect on alginate biosynthesis, which is important for the mucoid phenotype of *P. aeruginosa* colonies.^35^ Direct and indirect interactions reflect the importance of influential genes, not only to their specific targets, but the effects of their targets on the regulation of subsequent processes, triggering a more pleiotropic effect. If a perturbation is required, a hub can affect more than one pathway, resulting in undesired effects. However, one of the interconnected nodes related to that hub may be a candidate for a perturbation that results in the impairment or improvement of a specific pathway. Nevertheless, isolated hubs are equally important. In fact, they are related to processes such as zinc uptake (*np20*) and purine metabolism (PA4851_19380), which are fundamental to bacterial survival, but can be considered somewhat independent of other processes, and are only triggered under specific conditions.

Table II shows a comparison of network statistics between the CCBH4851 GRN and the GRN network published by Galán-Vásquez et al.^12^ One clear trend is that the CCBH4851 GRN represents a substantial improvement in terms of network completeness, since is includes more nodes, edges and network motifs. Other measures also reflect this improvement, such as the global clustering coefficient and the diameter of the network. Other comparisons between these networks are presented in Fig. 3. The charts and the data presented in Table II show an increase in the completeness and complexity of the CCBH4851 network, compared with the previous network, particularly when comparing clustering coefficients (Fig. 3E, F).

A concept addressed by Csermely,^36^ is the plasticity of networks. Plastic networks have some interesting characteristics, such as a diffuse core, overlapping modules, fewer hierarchies/more loops, large network entropy and origin dominance, leading to many attractors. Csermely states that biologically plastic networks should be targeted by a “central impact” directed at their hubs, bridges and bottlenecks, because if they are attacked on their periphery, the effect of the drug will never reach the centre of the network, due its efficient dissipation. For this reason, topological characteristics, such as connected components, motifs and hubs are important to help determine the best approach to disturb a network in a way that promotes the desired phenotype in the cell. Indeed, it is noteworthy that the total network entropy of the CCBH4851 GRN was greater than the *P. aeruginosa* GRN published in 2011 (Table II). Therefore, the CCBH4851 GRN had greater plasticity than the GRN described by Galán-Vásquez et al.^12^ This increased plasticity may be due to the increased size of the CCBH4851 GRN. Nevertheless, this observation may also be related to the fact that the CCBH4851 is an MDR strain, while the previous network is based mostly on *P. aeruginosa* PAO1. 

This reconstruction of the *P. aeruginosa* GRN contributes to an increase in our understanding of the behaviour of this bacterium. In future studies, we intend to construct a dynamic model of this network, aimed at assisting researchers working on experimental drug design and screening. This will enable the prediction of dynamic behaviour in order to better understand these bacteria and allow the simulation of normal and stress conditions, eventually leading to the discovery of new therapeutic targets and the development of new drugs to combat *P. aeruginosa* infections.
