# ERRATUM: Gene regulatory network inference and analysis of multidrug-resistant Pseudomonas aeruginosa

**DOI:** 10.1590/0074-02760190105ER

**Published:** 2020-06-12

 In the article **“Gene regulatory network inference and analysis of multidrug-resistant *Pseudomonas aeruginosa*”**, DOI number: 10.1590/0074-02760190105, published in *Mem Inst Oswaldo Cruz*, Rio de Janeiro, Vol. *114*: e190105, 2019:

**On page 7, Table I, where it reads:**

“PA4851_19380” 

It should read: 

“AL347_05155”

**On page 7, where it reads:**

“After pinpointing the hubs, an analysis was performed to determine whether they are interconnected (through direct or indirect interactions). Only two hubs were found to not be interconnected, *np20* and **PA4851_19380** (homologous to PA1520).” 

It should read: 

“After pinpointing the hubs, an analysis was performed to determine whether they are interconnected (through direct or indirect interactions). Only two hubs were found to not be interconnected, *np20* and **AL347_05155** (homologous to PA1520).”

**On page 10, where it reads:**

“In fact, they are related to processes such as zinc uptake (*np20*) and purine metabolism (**PA4851_19380**), which are fundamental to bacterial survival, but can be considered somewhat independent of other processes, and are only triggered under specific conditions.” 

It should read: 

“In fact, they are related to processes such as zinc uptake (*np20*) and purine metabolism (**AL347_05155**), which are fundamental to bacterial survival, but can be considered somewhat independent of other processes, and are only triggered under specific conditions.”

**On page 10, where it reads:**

“Table II shows a comparison of network statistics between the CCBH4851 GRN and the GRN network published by Galán-Vásquez et al. (12) One clear trend is that the CCBH4851 GRN represents a substantial improvement in terms of network completeness, since is includes more nodes, edges and network motifs.” 

It should read: 

“Table II shows a comparison of network statistics between the CCBH4851 GRN and the GRN network published by Galán-Vásquez et al. (12) One clear trend is that the CCBH4851 GRN represents a substantial improvement in terms of network completeness, since it includes more nodes, edges and network motifs.”

On page 4, Fig. 2 should be replaced by the figure below:

**Fig. 2:**
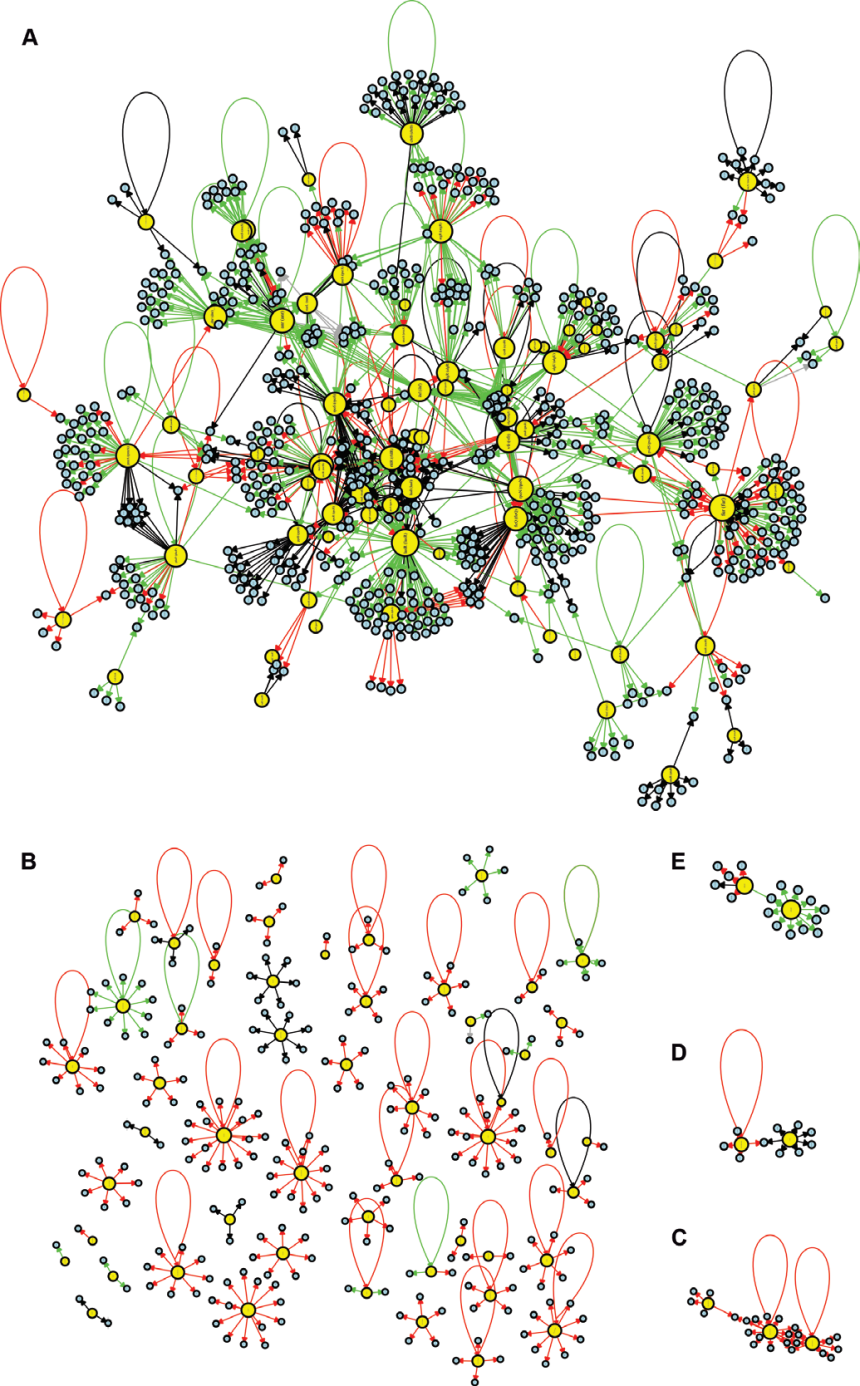
visualisation of the *Pseudomonas aeruginosa* CCBH4851 gene regulatory network (GRN). Yellow circles indicate regulatory genes, light blue circles indicate target genes (TGs), black lines indicate an unknown mode of regulation, green lines indicate activation, red lines indicate repression and grey lines indicate a dual mode of regulation. A: the GRN large highly connected network component; B: all regulatory and TGs that have no connections with the component depicted in A; C-E: clusters of lower connectivity compared to the component depicted in A. All figures are presented with higher resolution in the Supplementary data.

On page 8, Fig. 4 should be replaced by the figure below:

**Fig. 4:**
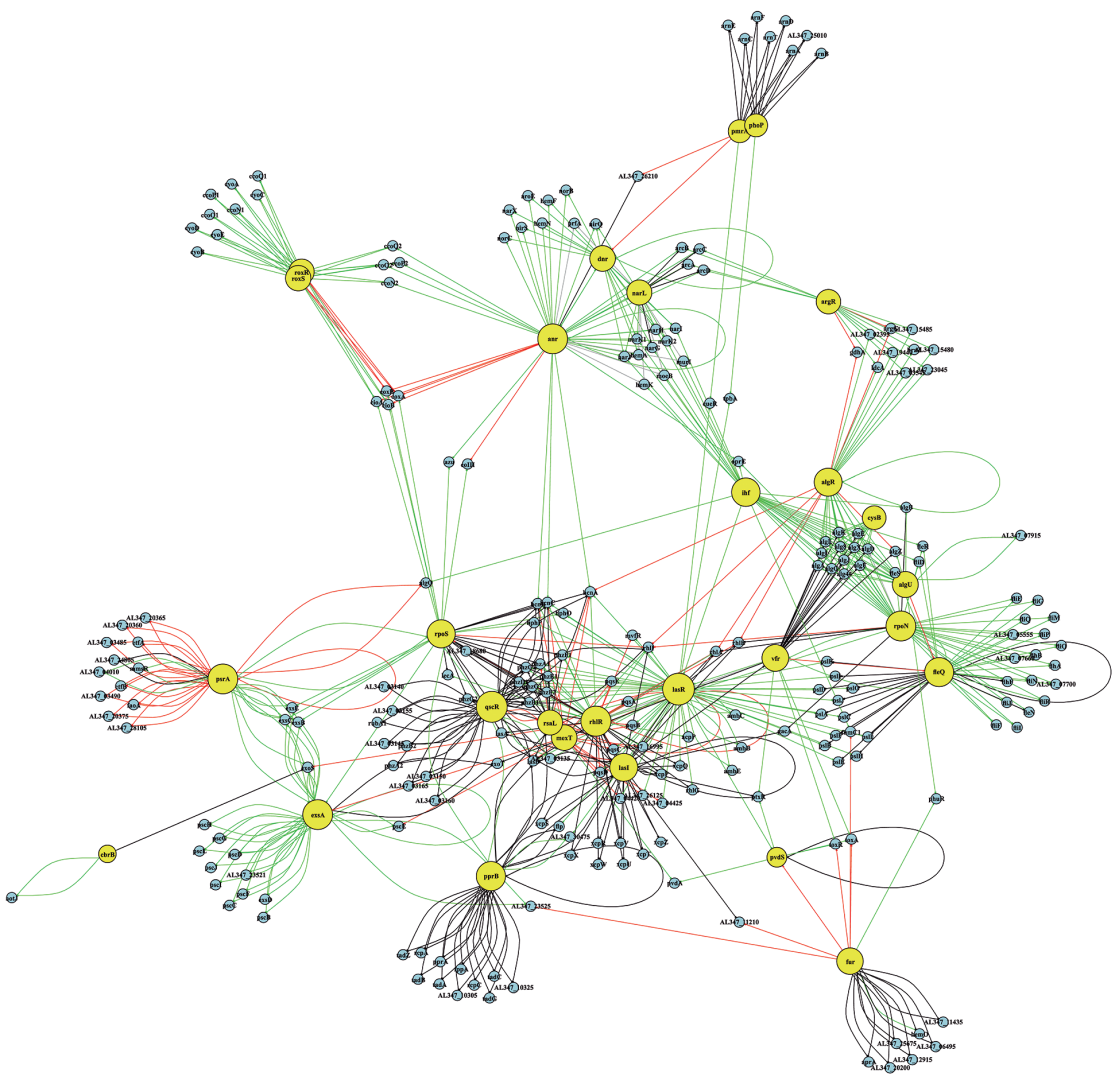
connectivity relationships among the 30 most influential hubs of the *Pseudomonas aeruginosa* CCBH4851 gene regulatory network. Yellow circles indicate regulatory genes considered hubs, light blue circles indicate target genes, black lines indicate an unknown mode of regulation, green lines indicate activation, red lines indicate repression and grey lines indicate a dual mode of regulation.

The Supplementary material were corrected and attached in the link below: 

https://memorias.ioc.fiocruz.br/images/revistas/2020/115/0105er_sd.xlsx

