# Supplementary material for: Gene regulatory network inference and analysis of multidrug-resistant Pseudomonas aeruginosa
Source: Mem Inst Oswaldo Cruz. 2019 Aug 5;114:e190105. doi: 10.1590/0074-02760190105 (PMC6684008; doi:10.1590/0074-02760190105)
Supplement: Supplementary file 1 [file 1678-8060-mioc-114-e190105-s.pdf]

Mem Inst Oswaldo Cruz, Rio de Janeiro, Vol. 114: e190105, 2019

NETWORK TABLE

Regulatory gene	Ortholog of the regulatory gene	Target gene	Ortholog of the target gene	Mode of regulation	Reference (PubMed)
aguR	aguR	aguA	aguA	-	27242034*
aguR	aguR	aguB	aguB	-	27242034*
algD	algD	algD	algD	?	18440972*
algQ	algQ	lasR	lasR	-	18440972*
algQ	algQ	rhlR	rhlR	-	18440972*
algQ	algQ	rpoD	rpoD	+	18440972*
algR	algR	alg44	alg44	+	22587778
algR	algR	alg8	alg8	+	22587778
algR	algR	algA	algA	+	22587778
algR	algR	algC	algC	+	22587778
algR	algR	algD	algD	+	22587778
algR	algR	algE	algE	+	22587778
algR	algR	algF	algF	+	22587778
algR	algR	algG	algG	+	22587778
algR	algR	algI	algI	+	22587778
algR	algR	algJ	algJ	+	22587778
algR	algR	algK	algK	+	22587778
algR	algR	algL	algL	+	22587778
algR	algR	algR	algR	+	22587778
algR	algR	algX	algX	+	22587778
algR	algR	algZ	algZ	-	22587778
algR	algR	argB	argB	+	22587778
algR	algR	argG	argG	-	22587778
algR	algR	braZ	braZ	+	22587778
algR	algR	gdhA	gdhA	-	22587778
algR	algR	hcnA	hcnA	-	22587778
algR	algR	PA4851_01650	PA0328	+	22587778
algR	algR	ldcA	ldcA	+	22587778
algR	algR	PA4851_17840	PA1819	+	22587778
algR	algR	PA4851_05160	PA3934	+	22587778
algR	algR	PA4851_29405	PA5152	+	22587778
algR	algR	PA4851_29410	PA5153	+	22587778
algR	algR	rhlA	rhlA	-	22587778
algR	algR	rhlB	rhlB	-	22587778
algR	algR	rhlI	rhlI	-	22587778
algR	algR	speA	speA	?	22587778
algR	algR	PA4851_16745	PA2042	+	18440972*
algU	algU	alg44	alg44	+	18440972*
algU	algU	alg8	alg8	+	18440972*
algU	algU	algA	algA	+	18974177*
algU	algU	algB	algB	+	18974177*
algU	algU	algD	algD	+	18440972*
algU	algU	algE	algE	+	18440972*
algU	algU	algF	algF	+	18440972*
algU	algU	algG	algG	+	18440972*

Regulatory gene	Ortholog of the regulatory gene	Target gene	Ortholog of the target gene	Mode of regulation	Reference (PubMed)
algU	algU	algI	algI	+	18440972*
algU	algU	algJ	algJ	+	18440972*
algU	algU	algK	algK	+	18440972*
algU	algU	algL	algL	+	18974177*
algU	algU	algR	algR	+	18974177*
algU	algU	algU	algU	+	18440972*
algU	algU	algX	algX	+	18974177*
algU	algU	algZ	algZ	+	22587778
algU	algU	bolA	bolA	+	22587778
algU	algU	dksA	dksA	+	22587778
algU	algU	fleQ	fleQ	-	21863142
algU	algU	PA4851_05320	ivy/ PA3902	+	22587778
algU	algU	lptA	lptA	+	22587778
algU	algU	PA4851_25200	lptB	+	22587778
algU	algU	mucA	mucA	+	22587778
algU	algU	mucB	mucB	+	22587778
algU	algU	oprF	oprF	+	22587778
algU	algU	osmC	osmC	+	22587778
algU	algU	PA4851_23315	PA0856	+	22587778
algU	algU	PA4851_19010	PA1592	+	22587778
algU	algU	PA4851_08455	PA3262	+	22587778
algU	algU	PA4851_05730	PA3819	+	22587778
algU	algU	PA4851_05070	PA3952	+	22587778
algU	algU	PA4851_30120	PA5291	+	22587778
algU	algU	phuR	phuR	+	22587778
algU	algU	rpoH	rpoH	+	22587778
algU	algU	PA4851_22040	PA1053	+	22587778
algU	algU	tal	tal	+	22587778
algU	algU	fusA	fusA	?	27242034*, 18974177*, 22587778
algU	algU	PA4851_22040	PA1053	+	27242034*
algU	algU	fliC	fliC	?	27242034*
algU	algU	osmE	osmE	?	27242034*
algU	algU	amrZ	amrZ	?	27242034*
algW	algW	algD	algD	+	12533483
algW	algW	algU	algU	+	12533483
algZ	algZ	alg44	alg44	+	18440972*
algZ	algZ	alg8	alg8	+	18440972*
algZ	algZ	algA	algA	+	18440972*
algZ	algZ	algD	algD	+	18440972*
algZ	algZ	algE	algE	+	18440972*
algZ	algZ	algF	algF	+	18440972*
algZ	algZ	algG	algG	+	18440972*
algZ	algZ	algI	algI	+	18440972*
algZ	algZ	algJ	algJ	+	18440972*
algZ	algZ	algK	algK	+	18440972*

Regulatory gene	Ortholog of the regulatory gene	Target gene	Ortholog of the target gene	Mode of regulation	Reference (PubMed)
algZ	algZ	algL	algL	+	18440972*
algZ	algZ	algX	algX	+	18440972*
algZ	algZ	algZ	algZ	-	18440972*
algZ	algZ	fleQ	fleQ	-	18440972*
ampR	ampR	ampC	ampC	+	22587778
ampR	ampR	lasA	lasA	-	22587778
ampR	ampR	lasB	lasB	+	22587778
ampR	ampR	lasI	lasI	-	22587778
ampR	ampR	lasR	lasR	-	22587778
ampR	ampR	poxB	poxB	-	22587778
ampR	ampR	rhlR	rhlR	+	22587778
anr	anr	anr	anr	+	27242034*
anr	anr	arcA	arcA	+	27242034*
anr	anr	arcB	arcB	+	27242034*
anr	anr	arcC	arcC	+	27242034*
anr	anr	azu	azu	+	27242034*
anr	anr	ccoN2	ccoN2	+	27242034*
anr	anr	ccoO2	ccoO2	+	27242034*
anr	anr	ccoP2	ccoP2	+	27242034*
anr	anr	ccoQ2	ccoQ2	+	27242034*
anr	anr	cioA	cioA	-	27242034*
anr	anr	cioB	cioB	-	27242034*
anr	anr	coIII	coIII	-	27242034*
anr	anr	coxA	coxA	-	27242034*
anr	anr	coxB	coxB	-	27242034*
anr	anr	dnr	dnr	+	27242034*
anr	anr	hcnA	hcnA	+	27242034*
anr	anr	hcnB	hcnB	+	27242034*
anr	anr	hcnC	hcnC	+	27242034*
anr	anr	hemA	hemA	+	27242034*
anr	anr	hemF	hemF	+	27242034*
anr	anr	hemK	hemK	d	27242034*
anr	anr	hemN	hemN	+	27242034*
anr	anr	moeB	moeB	d	27242034*
anr	anr	murI	murI	d	27242034*
anr	anr	narG	narG	+	27242034*
anr	anr	narH	narH	+	27242034*
anr	anr	narI	narI	+	27242034*
anr	anr	narJ	narJ	+	27242034*
anr	anr	narK1	narK1	+	27242034*
anr	anr	narK2	narK2	+	27242034*
anr	anr	narL	narL	+	27242034*
anr	anr	narX	narX	+	27242034*
anr	anr	nirQ	nirQ	+	27242034*
anr	anr	nirS	nirS	+	27242034*

Regulatory gene	Ortholog of the regulatory gene	Target gene	Ortholog of the target gene	Mode of regulation	Reference (PubMed)
anr	anr	norB	norB	+	27242034*
anr	anr	norC	norC	+	27242034*
anr	anr	oprE	oprE	+	27242034*
anr	anr	PA4851_02610	PA0521	+	27242034*
anr	anr	PA4851_02615	PA0522	+	27242034*
anr	anr	PA4851_02635	PA0526	?	27242034*
anr	anr	PA4851_05190	PA3928	?	27242034*
anr	anr	PA4851_24640	PA4352	+	27242034*
anr	anr	prfA	prfA	d	27242034*
anr	anr	arcD	arcD	+	27242034*
anr	anr	aroE	aroE	+	27242034*
anr	anr	PA4851_08205	PA3309	?	27242034*
anr	anr	aer	aer	?	27242034*
anr	anr	PA4851_16350	PA2127	?	27242034*
anr	anr	PA4851_16360	PA2126	?	27242034*
argR	argR	ldcA	ldcA	+	18440972*
argR	argR	PA4851_17840	PA1819	+	18440972*
argR	argR	aotJ	aotJ	+	18440972*
argR	argR	aotM	aotM	+	18440972*
argR	argR	aotP	aotP	+	18440972*
argR	argR	aotQ	aotQ	+	18440972*
argR	argR	arcA	arcA	+	18440972*
argR	argR	arcB	arcB	+	18440972*
argR	argR	arcC	arcC	+	18440972*
argR	argR	arcD	arcD	+	18440972*
argR	argR	argF	argF	-	18440972*
argR	argR	argG	argG	-	18440972*
argR	argR	argR	argR	+	18440972*
argR	argR	aruC	aruC	+	18440972*
argR	argR	aruF	aruF	+	18440972*
argR	argR	braZ	braZ	+	18440972*
argR	argR	carA	carA	-	18440972*
argR	argR	carB	carB	-	18440972*
argR	argR	gdhA	gdhA	-	18440972*
argR	argR	gdhB	gdhB	+	18440972*
argR	argR	gltB	gltB	-	18440972*
argR	argR	gltD	gltD	-	18440972*
argR	argR	greA	greA	-	18440972*
argR	argR	PA4851_01650	PA0328	+	18440972*
argR	argR	PA4851_23090	PA0900	+	18440972*
argR	argR	PA4851_16745	PA2042	+	18440972*
argR	argR	PA4851_07145	PA3538	-	18440972*
argR	argR	PA4851_05160	PA3934	+	18440972*
argR	argR	PA4851_27360	PA4754	-	18440972*
argR	argR	PA4851_27375	PA4757	-	18440972*

Regulatory gene	Ortholog of the regulatory gene	Target gene	Ortholog of the target gene	Mode of regulation	Reference (PubMed)
argR	argR	PA4851_29405	PA5152	+	18440972*
argR	argR	PA4851_29410	PA5153	+	18440972*
argR	argR	PA4851_29415	PA5154	+	18440972*
argR	argR	PA4851_29420	PA5155	+	18440972*
argR	argR	PA4851_23135	PA0891	+	18440972*
argR	argR	aruG	aruG	+	18440972*
argR	argR	aruB	aruB	+	18440972*
argR	argR	aruD	aruD	+	18440972*
argR	argR	aruE	aruE	+	18440972*
atuR	atuR	atuD	atuD	?	27242034*
atuR	atuR	atuE	atuE	?	27242034*
atuR	atuR	atuF	atuF	?	27242034*
atuR	atuR	atuG	atuG	?	27242034*
atuR	atuR	atuA	atuA	?	27242034*
atuR	atuR	atuB	atuB	?	27242034*
atuR	atuR	atuC	atuC	?	27242034*
atuR	atuR	atuH	atuH	?	27242034*
bexR	bexR	aprA	aprA	+	20041030
bexR	bexR	bexR	bexR	+	20041030
bexR	bexR	PA4851_02865	PA0572	+	20041030
bexR	bexR	PA4851_21290	PA1202	+	20041030
bexR	bexR	PA4851_21285	PA1203	+	20041030
bexR	bexR	PA4851_21280	PA1204	+	20041030
bexR	bexR	PA4851_21275	PA1205	+	20041030
birA	birA	bioB	bioB	-	27242034*
birA	birA	bioF	bioF	-	27242034*
birA	birA	PA4851_02515	bioH	-	27242034*
birA	birA	bioC	bioC	-	27242034*
birA	birA	bioD	bioD	-	27242034*
brlR	brlR	brlR	brlR	+	29967320
brlR	brlR	mexA	mexA	+	23687276
brlR	brlR	mexE	mexE	+	23687276
brlR	brlR	oprH	oprH	-	23935054
cbrB	cbrB	aotJ	aotJ	+	22587778
cbrB	cbrB	aotM	aotM	+	22587778
cbrB	cbrB	aotP	aotP	+	22587778
cbrB	cbrB	aotQ	aotQ	+	22587778
cbrB	cbrB	cbrB	cbrB	+	22587778
cbrB	cbrB	spuA	spuA	+	22587778
cbrB	cbrB	spuB	spuB	+	22587778
cbrB	cbrB	spuC	spuC	+	22587778
cbrB	cbrB	spuD	spuD	+	22587778
cbrB	cbrB	spuE	spuE	+	22587778
cbrB	cbrB	spuF	spuF	+	22587778
cbrB	cbrB	spuG	spuG	+	22587778

Regulatory gene	Ortholog of the regulatory gene	Target gene	Ortholog of the target gene	Mode of regulation	Reference (PubMed)
cbrB	cbrB	spuH	spuH	+	22587778
cbrB	cbrB	spuI	spuI	+	22587778
cbrB	cbrB	PA4851_29115	PA5104	?	27242034*
cbrB	cbrB	PA4851_29100	PA5101	?	27242034*
cbrB	cbrB	PA4851_29110	PA5103	?	27242034*
cbrB	cbrB	PA4851_29105	PA5102	?	27242034*
cbrB	cbrB	aotJ	aotJ	+	27242034*
cbrB	cbrB	PA4851_29090	PA5099	?	27242034*
cbrB	cbrB	PA4851_29060	PA5093	?	27242034*
cbrB	cbrB	PA4851_29080	PA5097	?	27242034*
cbrB	cbrB	PA4851_29075	PA5096	?	27242034*
cbrB	cbrB	PA4851_29070	PA5095	?	27242034*
cbrB	cbrB	PA4851_29065	PA5094	?	27242034*
cbrB	cbrB	hutU	hutU	?	27242034*
cbrB	cbrB	hutI	hutI	?	27242034*
cbrB	cbrB	hutH	hutH	?	27242034*
cbrB	cbrB	exoS	exoS	?	27242034*
cbrB	cbrB	hutG	hutG	?	27242034*
cbrB	cbrB	hutC	hutC	?	27242034*
cdhR	PSPA7_RS29445	PA4851_30635	PSPA7_RS29440 (PSPA7_6174)	?	27242034*
cdhR	PSPA7_RS29445	cdhC	PSPA7_RS29435 (PSPA7_6173)	?	27242034*
cifR	cifR	morB	morB	?	27242034*
cifR	cifR	PA4851_10135	PA2933	?	27242034*
cifR	cifR	cif	cif	?	27242034*
copR	copR	PA4851_13705	PA2524	?	27242034*
copR	copR	PA4851_13710	PA2523	?	27242034*
copR	copR	czcB	czcB	?	27242034*
copR	copR	czcC	czcC	?	27242034*
copR	copR	czcA	czcA	?	27242034*
copR	copR	ptrA	ptrA	?	27242034*
crc	crc	zwf	zwf	?	30429516
crc	crc	bkdB	bkdB	?	30429516
crc	crc	mtlD	mtlD	?	30429516
crc	crc	bkdA1	bkdA1	?	30429516
crc	crc	bkdA2	bkdA2	?	30429516
crc	crc	pilB	pilB	?	27242034*
crc	crc	lpdV	lpdV	?	27242034*
CueR	CueR	PA4851_05230	PA3920	+	24175918*
CueR	CueR	PA4851_07220	PA3523	+	24175918*
CueR	CueR	PA4851_07225	PA3522	+	24175918*
CueR	CueR	PA4851_07230	PA3521	+	24175918*
cysB	cysB	alg44	alg44	+	22587778
cysB	cysB	alg8	alg8	+	22587778
cysB	cysB	algA	algA	+	22587778
cysB	cysB	algD	algD	+	22587778

Regulatory gene	Ortholog of the regulatory gene	Target gene	Ortholog of the target gene	Mode of regulation	Reference (PubMed)
cysB	cysB	algE	algE	+	22587778
cysB	cysB	algF	algF	+	22587778
cysB	cysB	algG	algG	+	22587778
cysB	cysB	algI	algI	+	22587778
cysB	cysB	algJ	algJ	+	22587778
cysB	cysB	algK	algK	+	22587778
cysB	cysB	algL	algL	+	22587778
cysB	cysB	algX	algX	+	22587778
cysB	cysB	aruG	aruG	+	22587778
cysB	cysB	aruB	aruB	+	22587778
cysB	cysB	aruC	aruC	+	22587778
cysB	cysB	cysB	cysB	-	22587778
cysB	cysB	PA4851_14870	PA2355	+	22587778
cysB	cysB	msuD	msuD	+	22587778
cysB	cysB	msuE	msuE	+	22587778
cysB	cysB	PA4851_00950	PA0185	?	27242034*
cysB	cysB	atsA	atsA	?	27242034*
desT	desT	fabA	fabA	-	27242034*
desT	desT	PA4851_28040	PA4889	-	27242034*
desT	desT	desB	desB	-	27242034*
desT	desT	desT	desT	?	27242034*
dnr	dnr	anr	anr	+	22587778
dnr	dnr	aroE	aroE	+	22587778
dnr	dnr	dnr	dnr	+	22587778
dnr	dnr	hemA	hemA	+	22587778
dnr	dnr	hemF	hemF	+	22587778
dnr	dnr	hemN	hemN	+	22587778
dnr	dnr	narG	narG	+	22587778
dnr	dnr	narH	narH	+	22587778
dnr	dnr	narI	narI	+	22587778
dnr	dnr	narJ	narJ	+	22587778
dnr	dnr	narK1	narK1	+	22587778
dnr	dnr	narK2	narK2	+	22587778
dnr	dnr	narL	narL	+	22587778
dnr	dnr	narX	narX	+	22587778
dnr	dnr	nirC	nirC	+	22587778
dnr	dnr	nirD	nirD	+	22587778
dnr	dnr	nirF	nirF	+	22587778
dnr	dnr	PA4851_02565	nirH	+	22587778
dnr	dnr	nirJ	nirJ	+	22587778
dnr	dnr	nirL	nirL	+	22587778
dnr	dnr	nirM	nirM	+	22587778
dnr	dnr	nirN	nirN	+	22587778
dnr	dnr	nirQ	nirQ	+	22587778
dnr	dnr	nirS	nirS	+	22587778

Regulatory gene	Ortholog of the regulatory gene	Target gene	Ortholog of the target gene	Mode of regulation	Reference (PubMed)
dnr	dnr	norB	norB	+	22587778
dnr	dnr	norC	norC	+	22587778
dnr	dnr	nosD	nosD	+	22587778
dnr	dnr	nosF	nosF	+	22587778
dnr	dnr	nosL	nosL	+	22587778
dnr	dnr	nosR	nosR	+	22587778
dnr	dnr	nosY	nosY	+	22587778
dnr	dnr	nosZ	nosZ	+	22587778
dnr	dnr	PA4851_02555	PA0510	+	22587778
dnr	dnr	PA4851_02570	PA0513	+	22587778
erbR	agmR	exaA	exaA	+	27242034*
erbR	agmR	exaB	exaB	+	27242034*
erbR	agmR	exaC	exaC	+	27242034*
erbR	agmR	eraS	exaD	+	27242034*
erbR	agmR	eraR	exaE	+	27242034*
erbR	agmR	pqqA	pqqA	+	27242034*
erbR	agmR	pqqB	pqqB	+	27242034*
erbR	agmR	pqqC	pqqC	+	27242034*
erbR	agmR	pqqD	pqqD	+	27242034*
erbR	agmR	pqqE	pqqE	+	27242034*
erbR	agmR	pqqH	pqqH	+	27242034*
exsA	exsA	exoS	exoS	+	18440972*
exsA	exsA	exsA	exsA	+	18440972*
exsA	exsA	exsB	exsB	+	18440972*
exsA	exsA	exsC	exsC	+	18440972*
exsA	exsA	exsD	exsD	+	18440972*
exsA	exsA	exsE	exsE	+	18440972*
exsA	exsA	PA4851_05610	PA3842	+	18440972*
exsA	exsA	PA4851_05605	PA3843	+	18440972*
exsA	exsA	pscB	pscB	+	18440972*
exsA	exsA	pscC	pscC	+	18440972*
exsA	exsA	pscD	pscD	+	18440972*
exsA	exsA	pscE	pscE	+	18440972*
exsA	exsA	pscF	pscF	+	18440972*
exsA	exsA	pscG	pscG	+	18440972*
exsA	exsA	pscH	pscH	+	18440972*
exsA	exsA	pscI	pscI	+	18440972*
exsA	exsA	pscJ	pscJ	+	18440972*
exsA	exsA	pscL	pscL	+	18440972*
exsA	exsA	exoS	exoS	+	27242034*
exsA	exsA	exoT	exoT	?	27242034*
exsD	exsD	exsA	exsA	-	27242034*
fleQ	fleQ	fleR	fleR	+	22587778
fleQ	fleQ	fleS	fleS	+	22587778
fleQ	fleQ	flhA	flhA	+	22587778

Regulatory gene	Ortholog of the regulatory gene	Target gene	Ortholog of the target gene	Mode of regulation	Reference (PubMed)
fleQ	fleQ	flhB	flhB	+	22587778
fleQ	fleQ	fliD	fliD	+	22587778
fleQ	fleQ	fliE	fliE	+	22587778
fleQ	fleQ	fliF	fliF	+	22587778
fleQ	fleQ	fliG	fliG	+	22587778
fleQ	fleQ	PA4851_21790	fliH	+	22587778
fleQ	fleQ	fliI	fliI	+	22587778
fleQ	fleQ	fliJ	fliJ	+	22587778
fleQ	fleQ	fliM	fliM	+	22587778
fleQ	fleQ	fliN	fliN	+	22587778
fleQ	fleQ	fliO	fliO	+	22587778
fleQ	fleQ	fliP	fliP	+	22587778
fleQ	fleQ	fliQ	fliQ	+	22587778
fleQ	fleQ	fliR	fliR	+	22587778
fleQ	fleQ	PA4851_21830	fliS	+	22587778
fleQ	fleQ	fur	fur	-	22587778
fleQ	fleQ	pelA	pelA	-	22587778
fleQ	fleQ	pelB	pelB	-	22587778
fleQ	fleQ	fleQ	fleQ	?	22587778
fleQ	fleQ	PA4851_19770	PA1442	+	22587778
fleQ	fleQ	PA4851_21825	PA1096	?	27242034*
fleQ	fleQ	pslI	pslI	?	27242034*
fleQ	fleQ	flhF	flhF	?	27242034*
fleQ	fleQ	pelE	pelE	?	27242034*
fleQ	fleQ	pelD	pelD	?	27242034*
fleQ	fleQ	pelG	pelG	?	27242034*
fleQ	fleQ	pelF	pelF	?	27242034*
fleQ	fleQ	pelC	pelC	?	27242034*
fleQ	fleQ	PA4851_14440	PA2441	?	27242034*
fleQ	fleQ	pslK	pslK	?	27242034*
fleQ	fleQ	pslJ	pslJ	?	27242034*
fleQ	fleQ	pslH	pslH	?	27242034*
fleQ	fleQ	pslL	pslL	?	27242034*
fleQ	fleQ	pslC	pslC	?	27242034*
fleQ	fleQ	pslB	pslB	?	27242034*
fleQ	fleQ	pslA	pslA	?	27242034*
fleQ	fleQ	pslG	pslG	?	27242034*
fleQ	fleQ	pslF	pslF	?	27242034*
fleQ	fleQ	pslE	pslE	?	27242034*
fleQ	fleQ	fleN	fleN	?	27242034*
fleQ	fleQ	pslD	pslD	?	27242034*
flgM	flgM	fliA	fliA	-	22587778
flgM	flgM	fliC	fliC	-	22587778
fliA	fliA	flgM	flgM	+	22587778
fliA	fliA	fliC	fliC	+	22587778

Regulatory gene	Ortholog of the regulatory gene	Target gene	Ortholog of the target gene	Mode of regulation	Reference (PubMed)
fliA	fliA	PA4851_19770	PA1442	+	22587778
fliA	fliA	PA4851_21840	PA1093	?	27242034*
fliA	fliA	PA4851_07990	PA3352	?	27242034*
fliA	fliA	fliA	fliA	?	27242034*
fpvR	fpvR	fpvI	fpvI	-	22587778
fpvR	fpvR	pvdS	pvdS	-	22587778
fruR	fruR	fruR	fruR	-	27242034*
fruR	fruR	fruK	fruK	-	27242034*
fruR	fruR	fruA	fruA	-	27242034*
fruR	fruR	fruI	fruI	-	27242034*
fur	fur	PA4851_25250	fagA	-	22587778
fur	fur	foxI	foxI	-	22587778
fur	fur	foxR	foxR	-	22587778
fur	fur	fptA	fptA	+	22587778
fur	fur	PA4851_03740	fptB	+	22587778
fur	fur	fpvR	fpvR	+	22587778
fur	fur	gbuR	gbuR	-	22587778
fur	fur	hasAp_P	hasAp_P	+	22587778
fur	fur	hasAp_N	hasAp_N	-	22587778
fur	fur	icmP	icmP	+	22587778
fur	fur	oprL	oprL	-	22587778
fur	fur	motD	motD	-	22587778
fur	fur	PA4851_00380	PA0071	-	22587778
fur	fur	PA4851_00385	PA0072	-	22587778
fur	fur	PA4851_22680	PA0929	-	22587778
fur	fur	PA4851_22675	PA0930	-	22587778
fur	fur	PA4851_20800	PA1300	-	22587778
fur	fur	PA4851_20795	PA1301	-	22587778
fur	fur	PA4851_16785	PA2033	+	22587778
fur	fur	PA4851_16780	PA2034	+	22587778
fur	fur	PA4851_07685	PA3409	+	22587778
fur	fur	PA4851_07185	PA3530	+	22587778
fur	fur	PA4851_05335	PA3899	-	22587778
fur	fur	PA4851_05330	PA3900	-	22587778
fur	fur	PA4851_25230	PA4467	-	22587778
fur	fur	PA4851_25240	PA4469	-	22587778
fur	fur	PA4851_25475	PA4516	+	22587778
fur	fur	PA4851_26350	PA4570	+	22587778
fur	fur	PA4851_28070	PA4895	+	22587778
fur	fur	PA4851_28075	PA4896	+	22587778
fur	fur	PA4851_29740	PA5216	+	22587778
fur	fur	PA4851_29745	PA5217	+	22587778
fur	fur	pchA	pchA	-	22587778
fur	fur	pchB	pchB	-	22587778
fur	fur	pchC	pchC	-	22587778

Regulatory gene	Ortholog of the regulatory gene	Target gene	Ortholog of the target gene	Mode of regulation	Reference (PubMed)
fur	fur	pchD	pchD	-	22587778
fur	fur	pchE	pchE	-	22587778
fur	fur	pchF	pchF	-	22587778
fur	fur	pchR	pchR	-	22587778
fur	fur	pfeR	pfeR	-	22587778
fur	fur	phuT	phuT	+	22587778
fur	fur	hemO	hemO	+	22587778
fur	fur	PA4851_25470	piuC	+	22587778
fur	fur	pvdQ	pvdQ	+	22587778
fur	fur	pvdS	pvdS	-	22587778
fur	fur	rplA	rplA	+	22587778
fur	fur	rplJ	rplJ	+	22587778
fur	fur	rplL	rplL	+	22587778
fur	fur	sodM	sodA	-	22587778
fur	fur	tolA	tolA	-	22587778
fur	fur	tolB	tolB	-	22587778
fur	fur	tolQ	tolQ	-	22587778
fur	fur	tolR	tolR	-	22587778
fur	fur	toxA	toxA	-	22587778
fur	fur	PA4851_22485	ybgC	?	22587778
fur	fur	PA4851_27090	PSPA7_RS25870	-	22587778
fur	fur	fpvI	fpvI	-	22587778
fur	fur	fumC1	fumC1	-	22587778
fur	fur	hasR	hasR	+	22587778
fur	fur	PA4851_05610	PA3842	-	22587778
fur	fur	phuR	phuR	+	22587778
fur	fur	PA4851_27105	PA4709	+	22587778
fur	fur	PA4851_27095	PA4707	+	22587778
fur	fur	PA4851_27085	PA4705	+	22587778
fur	fur	toxR	toxR	-	22587778
fur	fur	PA4851_26895	PA4675	?	27242034*
fur	fur	PA4851_20690	PA1322	?	27242034*
fur	fur	aprA	aprA	?	27242034*
fur	fur	PA4851_25460	PA4513	?	27242034*
fur	fur	PA4851_07680	PA3410	?	27242034*
fur	fur	PA4851_02375	PA0473	?	27242034*
fur	fur	PA4851_26895	PA4675	?	27242034*
fur	fur	PA4851_20690	PA1322	?	27242034*
fur	fur	aprA	aprA	?	27242034*
fur	fur	PA4851_25460	PA4513	?	27242034*
fur	fur	PA4851_07680	PA3410	?	27242034*
fur	fur	hemO	hemO	?	27242034*
fur	fur	PA4851_02375	PA0473	?	27242034*
gacA	gacA	hcnA	hcnA	+	22587778
gacA	gacA	hcnB	hcnB	+	22587778

Regulatory gene	Ortholog of the regulatory gene	Target gene	Ortholog of the target gene	Mode of regulation	Reference (PubMed)
gacA	gacA	hcnC	hcnC	+	22587778
gacA	gacA	lasR	lasR	+	22587778
gacA	gacA	PA4851_02645	RsmY	+	22587778
gacA	gacA	PA4851_06725	RsmZ	+	22587778
gacA	gacA	lasI	lasI	?	22587778
gacS	gacS	PA4851_02645	RsmY	+	27242034*
gacS	gacS	PA4851_06725	RsmZ	+	27242034*
gbdR	PSPA7_RS29400	PA4851_30680	PSPA7_RS29485 (PSPA7_6184)	?	27242034*
gbdR	PSPA7_RS29400	soxB	soxB	?	27242034*
gbdR	PSPA7_RS29400	soxA	soxA	?	27242034*
gbdR	PSPA7_RS29400	soxG	soxG	?	27242034*
gbdR	PSPA7_RS29400	dgcA	PSPA7_RS29490 (PSPA7_6185)	?	27242034*
gbdR	PSPA7_RS29400	gbcA	PSPA7_RS29550 (PSPA7_6197)	?	27242034*
gbdR	PSPA7_RS29400	PA4851_30675	PSPA7_RS29480 (PSPA7_6182)	?	27242034*
gbuR	gbuR	glmS	glmS	?	27242034*
gbuR	gbuR	gpuP	gpuP	+	27242034*
gbuR	gbuR	gpuA	gpuA	d	27242034*
gbuR	gbuR	ptxS	ptxS	+	27242034*
gbuR	gbuR	gbuA	gbuA	d	27242034*
gbuR	gbuR	glpR	glpR	+	27242034*
GlcC	GlcC	glcD	glcD	-	27242034*
GlcC	GlcC	glcE	glcE	-	27242034*
GlcC	GlcC	glcF	glcF	-	27242034*
GlcC	GlcC	PA4851_30430	PA5352	-	27242034*
GlcC	GlcC	glcC	glcC	-	27242034*
glmR	PSPA7_RS30295	glmS	glmS	?	18440972*
glpR	glpR	erbR	agmR	+	18440972*, 22587778
glpR	glpR	glpD	glpD	-	22587778, 18440972*
glpR	glpR	glpF	glpF	-	22587778, 18974177*
glpR	glpR	PA4851_06940	glpK1	?	22587778, 18974177*
glpR	glpR	glpT	glpT	-	22587778, 18974177*
glpR	glpR	himA	himA	-	22587778, 18974177*
gntR	gntR	PA4851_15035	PA2322	-	27242034*
gntR	gntR	PA4851_15040	PA2321	-	27242034*
gntR	gntR	gntR	gntR	-	27242034*
gpuR	gpuR	gpuA	gpuA	+	24175918*
gpuR	gpuR	gpuP	gpuP	+	24175918*
gpuR	gpuR	gpuR	gpuR	+	24175918*
HutC	HutC	hutC	hutC	-	27242034*
HutC	HutC	PA4851_29115	PA5104	-	27242034*
HutC	HutC	PA4851_29125	PA5106	-	27242034*
HutC	HutC	hutU	hutU	-	27242034*
HutC	HutC	PA4851_29090	PA5099	-	27242034*
HutC	HutC	hutH	hutH	-	27242034*
HutC	HutC	PA4851_29080	PA5097	-	27242034*

Regulatory gene	Ortholog of the regulatory gene	Target gene	Ortholog of the target gene	Mode of regulation	Reference (PubMed)
HutC	HutC	PA4851_29075	PA5096	-	27242034*
HutC	HutC	PA4851_29070	PA5095	-	27242034*
HutC	HutC	PA4851_29065	PA5094	-	27242034*
HutC	HutC	PA4851_29060	PA5093	-	27242034*
HutC	HutC	hutI	hutI	-	27242034*
HutC	HutC	hutG	hutG	-	27242034*
ihf	ihf	alg44	alg44	+	22587778
ihf	ihf	alg8	alg8	+	22587778
ihf	ihf	algA	algA	+	22587778
ihf	ihf	algB	algB	+	22587778
ihf	ihf	algD	algD	+	22587778
ihf	ihf	algE	algE	+	22587778
ihf	ihf	algF	algF	+	22587778
ihf	ihf	algG	algG	+	22587778
ihf	ihf	algI	algI	+	22587778
ihf	ihf	algJ	algJ	+	22587778
ihf	ihf	algK	algK	+	22587778
ihf	ihf	algL	algL	+	22587778
ihf	ihf	algX	algX	+	22587778
ihf	ihf	fleR	fleR	+	22587778
ihf	ihf	fleS	fleS	+	22587778
ihf	ihf	fliD	fliD	+	22587778
ihf	ihf	fumC1	fumC1	+	22587778
ihf	ihf	hemA	hemA	+	22587778
ihf	ihf	hemK	hemK	+	22587778
ihf	ihf	lasR	lasR	+	22587778
ihf	ihf	moeB	moeB	+	22587778
ihf	ihf	murI	murI	+	22587778
ihf	ihf	narG	narG	+	22587778
ihf	ihf	narH	narH	+	22587778
ihf	ihf	narI	narI	+	22587778
ihf	ihf	narJ	narJ	+	22587778
ihf	ihf	narK1	narK1	+	22587778
ihf	ihf	narK2	narK2	+	22587778
ihf	ihf	oprE	oprE	+	22587778
ihf	ihf	algQ	algQ	+	22587778
iscR	iscR	iscR	iscR	-	27242034*
iscR	iscR	iscS	iscS	-	27242034*
iscR	iscR	iscU	iscU	-	27242034*
iscR	iscR	iscA	iscA	-	27242034*
iscR	iscR	hscB	hscB	-	27242034*
iscR	iscR	hscA	hscA	-	27242034*
iscR	iscR	fdx2	fdx2	-	27242034*
iscR	iscR	PA4851_05785	PA3808	-	27242034*
iscR	iscR	PA4851_03365	PA0665	-	27242034*

Regulatory gene	Ortholog of the regulatory gene	Target gene	Ortholog of the target gene	Mode of regulation	Reference (PubMed)
lasI	lasI	rsaL	rsaL	?	22587778, 27242034*
lasI	lasI	xcpP	xcpP	?	22587778, 27242034*
lasI	lasI	lasB	lasB	?	22587778, 27242034*
lasI	lasI	lasI	lasI	?	22587778, 27242034*
lasI	lasI	gacA	gacA	?	27242034*
lasI	lasI	xcpW	xcpW	?	27242034*
lasI	lasI	xcpV	xcpV	?	27242034*
lasI	lasI	rsaL	rsaL	?	27242034*
lasI	lasI	xcpT	xcpT	?	27242034*
lasI	lasI	xcpS	xcpS	?	27242034*
lasI	lasI	xcpR	xcpR	?	27242034*
lasI	lasI	xcpQ	xcpQ	?	27242034*
lasI	lasI	xcpP	xcpP	?	27242034*
lasI	lasI	xcpZ	xcpZ	?	27242034*
lasI	lasI	xcpX	xcpX	?	27242034*
lasI	lasI	qscR	qscR	?	27242034*
lasI	lasI	lasR	lasR	?	27242034*
lasI	lasI	rhlB	rhlB	?	27242034*
lasI	lasI	rhlA	rhlA	?	27242034*
lasI	lasI	xcpU	xcpU	?	27242034*
lasI	lasI	rhlI	rhlI	?	27242034*
lasI	lasI	lasA	lasA	?	27242034*
lasI	lasI	lasB	lasB	?	27242034*
lasI	lasI	rhlR	rhlR	?	27242034*
lasI	lasI	ampR	ampR	?	27242034*
lasI	lasI	lasI	lasI	?	27242034*
lasI	lasI	fagA	fagA	?	27242034*
lasI	lasI	PA4851_25240	PA4469	?	27242034*
lasI	lasI	ptxR	ptxR	?	27242034*
lasI	lasI	pprB	pprB	?	27242034*
lasI	lasI	eta	eta	?	27242034*
lasI	lasI	vfr	vfr	?	27242034*
lasR	lasR	acpP	acpP	+	22587778, 27242034*
lasR	lasR	PA4851_17590	PA1869	+	22587778, 27242034*
lasR	lasR	aprD	aprD	+	22587778, 27242034*
lasR	lasR	aprE	aprE	+	22587778, 27242034*
lasR	lasR	aprF	aprF	+	22587778, 27242034*
lasR	lasR	PA4851_21075	aprX	+	22587778, 27242034*
lasR	lasR	bphO	bphO	+	22587778, 27242034*
lasR	lasR	bphP	bphP	+	22587778, 27242034*
lasR	lasR	PA4851_08120	PA3326	+	22587778, 27242034*
lasR	lasR	flp	flp	+	22587778, 27242034*
lasR	lasR	hcnA	hcnA	+	22587778, 27242034*
lasR	lasR	hcnB	hcnB	+	22587778, 27242034*
lasR	lasR	hcnC	hcnC	+	22587778, 27242034*

Regulatory gene	Ortholog of the regulatory gene	Target gene	Ortholog of the target gene	Mode of regulation	Reference (PubMed)
lasR	lasR	PA4851_17365	hvn	+	22587778, 27242034*
lasR	lasR	PA4851_31115	kinB	+	22587778, 27242034*
lasR	lasR	kynB	kynB	+	22587778, 27242034*
lasR	lasR	lasB	lasB	+	22587778, 27242034*
lasR	lasR	lasI	lasI	+	22587778, 27242034*
lasR	lasR	mexR	mexR	+	22587778, 27242034*
lasR	lasR	mvfR	mvfR	+	22587778, 27242034*
lasR	lasR	nuh	nuh	+	22587778, 27242034*
lasR	lasR	PA4851_00165	PA0027	+	22587778, 27242034*
lasR	lasR	PA4851_00170	PA0028	+	22587778, 27242034*
lasR	lasR	PA4851_00635	PA0122	+	22587778, 27242034*
lasR	lasR	PA4851_00745	PA0144	+	22587778, 27242034*
lasR	lasR	PA4851_02865	PA0572	+	22587778, 27242034*
lasR	lasR	PA4851_23570	PA0805	+	22587778, 27242034*
lasR	lasR	PA4851_23320	PA0855	+	22587778, 27242034*
lasR	lasR	PA4851_21505	PA1159	+	22587778, 27242034*
lasR	lasR	PA4851_19930	PA1419	+	22587778, 27242034*
lasR	lasR	PA4851_18685	PA1656	+	22587778, 27242034*
lasR	lasR	PA4851_18680	PA1657	+	22587778, 27242034*
lasR	lasR	PA4851_18675	PA1658	+	22587778, 27242034*
lasR	lasR	PA4851_18670	PA1659	+	22587778, 27242034*
lasR	lasR	ambE	ambE	+	22587778, 27242034*
lasR	lasR	ambD	ambD	+	22587778, 27242034*
lasR	lasR	ambC	ambC	+	22587778, 27242034*
lasR	lasR	ambB	ambB	+	22587778, 27242034*
lasR	lasR	PA4851_12265	PA2588	+	22587778, 27242034*
lasR	lasR	PA4851_12250	PA2591	+	22587778, 27242034*
lasR	lasR	PA4851_10105	PA2939	+	22587778, 27242034*
lasR	lasR	PA4851_07160	PA3535	+	22587778, 27242034*
lasR	lasR	PA4851_05310	PA3904	+	22587778, 27242034*
lasR	lasR	PA4851_05305	PA3905	+	22587778, 27242034*
lasR	lasR	PA4851_05300	PA3906	+	22587778, 27242034*
lasR	lasR	PA4851_05295	PA3907	+	22587778, 27242034*
lasR	lasR	PA4851_05290	PA3908	+	22587778, 27242034*
lasR	lasR	PA4851_26905	PA4677	+	22587778, 27242034*
lasR	lasR	cueR	cueR	+	22587778, 27242034*
lasR	lasR	PA4851_29555	PA5181	+	22587778, 27242034*
lasR	lasR	PA4851_29580	PA5184	+	22587778, 27242034*
lasR	lasR	PA4851_29815	PA5230	+	22587778, 27242034*
lasR	lasR	PA4851_29820	PA5231	+	22587778, 27242034*
lasR	lasR	PA4851_29825	PA5232	+	22587778, 27242034*
lasR	lasR	phnC	phnC	+	22587778, 27242034*
lasR	lasR	phzA1	phzA1	+	22587778, 27242034*
lasR	lasR	phzB1	phzB1	+	22587778, 27242034*
lasR	lasR	phzC1	phzC1	+	22587778, 27242034*

Regulatory gene	Ortholog of the regulatory gene	Target gene	Ortholog of the target gene	Mode of regulation	Reference (PubMed)
lasR	lasR	phzD1	phzD1	+	22587778, 27242034*
lasR	lasR	phzE1	phzE1	+	22587778, 27242034*
lasR	lasR	phzF1	phzF1	+	22587778, 27242034*
lasR	lasR	phzG1	phzG1	+	22587778, 27242034*
lasR	lasR	plcB	plcB	+	22587778, 27242034*
lasR	lasR	pqsA	pqsA	+	22587778, 27242034*
lasR	lasR	pqsB	pqsB	+	22587778, 27242034*
lasR	lasR	pqsC	pqsC	+	22587778, 27242034*
lasR	lasR	pqsD	pqsD	+	22587778, 27242034*
lasR	lasR	pqsE	pqsE	+	22587778, 27242034*
lasR	lasR	pqsH	pqsH	+	22587778, 27242034*
lasR	lasR	pslA	pslA	+	22587778, 27242034*
lasR	lasR	pslB	pslB	+	22587778, 27242034*
lasR	lasR	pslC	pslC	+	22587778, 27242034*
lasR	lasR	pslD	pslD	+	22587778, 27242034*
lasR	lasR	pslE	pslE	+	22587778, 27242034*
lasR	lasR	pslF	pslF	+	22587778, 27242034*
lasR	lasR	pslG	pslG	+	22587778, 27242034*
lasR	lasR	pslH	pslH	+	22587778, 27242034*
lasR	lasR	pslI	pslI	+	22587778, 27242034*
lasR	lasR	pslJ	pslJ	+	22587778, 27242034*
lasR	lasR	pslK	pslK	+	22587778, 27242034*
lasR	lasR	pslL	pslL	+	22587778, 27242034*
lasR	lasR	pvdS	pvdS	+	22587778, 27242034*
lasR	lasR	PA4851_17450	PA1897	+	22587778, 27242034*
lasR	lasR	rhlG	rhlG	+	22587778, 27242034*
lasR	lasR	rhlI	rhlI	+	22587778, 27242034*
lasR	lasR	rhlR	rhlR	+	22587778, 27242034*
lasR	lasR	rsaL	rsaL	+	22587778, 27242034*
lasR	lasR	tpbA	tpbA	+	22587778, 27242034*
lasR	lasR	xcpP	xcpP	+	22587778, 27242034*
lasR	lasR	xcpQ	xcpQ	+	22587778, 27242034*
lasR	lasR	ambE	ambE	+	27242034*
lasR	lasR	ambB	ambB	+	27242034*
lasR	lasR	ambC	ambC	+	27242034*
lasR	lasR	phzF2	phzF2	+	27242034*
lasR	lasR	rhlA	rhlA	?	27242034*
lasR	lasR	kynU	kynU	?	27242034*
lasR	lasR	rhlB	rhlB	?	27242034*
lasR	lasR	PA4851_12245	PA2592	?	27242034*
lasR	lasR	qteE	qteE	?	27242034*
lexA	lexA	PA4851_00370	PA0069	-	27242034*
lexA	lexA	PA4851_22720	PA0922	-	27242034*
lexA	lexA	PA4851_24245	PA0671	-	27242034*
lexA	lexA	PA4851_24250	PA0670	-	27242034*

Regulatory gene	Ortholog of the regulatory gene	Target gene	Ortholog of the target gene	Mode of regulation	Reference (PubMed)
lexA	lexA	PA4851_24255	PA0669	-	27242034*
lexA	lexA	lexA	lexA	-	27242034*
lexA	lexA	PA4851_09750	PA3008	-	27242034*
lexA	lexA	recN	recN	-	27242034*
lexA	lexA	recA	recA	-	27242034*
lexA	lexA	PA4851_06755	PA3616	-	27242034*
lexA	lexA	PA4851_22080	PA1045	-	27242034*
lexA	lexA	PA4851_17610	PA1865	-	27242034*
lexA	lexA	PA4851_17605	PA1866	-	27242034*
lexA	lexA	PA4851_15200	PA2288	-	27242034*
lexA	lexA	PA4851_07665	PA3413	-	27242034*
metR	metR	atuF	atuF	+	27242034*, 18974177*, 22587778
metR	metR	metH	metH	+	27242034*, 18974177*, 22587778
metR	metR	ntrC	ntrC	-	27242034*, 18974177*, 22587778
metR	metR	PA4851_03205	PA0633	-	27242034*, 18974177*, 22587778
metR	metR	PA4851_23355	PA0848	-	27242034*, 18974177*, 22587778
metR	metR	PA4851_04120	PA4144	+	27242034*, 18974177*, 22587778
metR	metR	PA4851_03725	PA4223	+	27242034*, 18974177*, 22587778
metR	metR	pvdL	pvdL	+	27242034*, 18974177*, 22587778
metR	metR	pvdH	pvdH	+	27242034*, 18974177*, 22587778
metR	metR	metH	metH	-	27242034*, 18974177*, 22587778
metR	metR	metR	metR	-	27242034*, 18974177*, 22587778
metR	metR	metE	metE	-	27242034*, 18974177*, 22587778
mexR	mexR	mexA	mexA	-	27242034*, 18974177*, 22587778
mexR	mexR	mexB	mexB	-	27242034*, 18974177*, 22587778
mexR	mexR	mexT	mexT	-	27242034*, 18974177*, 22587778
mexR	mexR	nalC	nalC	-	27242034*, 18974177*, 22587778
mexR	mexR	oprM	oprM	-	27242034*, 18974177*, 22587778
mexT	mexT	cbpD	cbpD	-	27242034*, 18974177*, 22587778
mexT	mexT	exsA	exsA	-	27242034*, 18974177*, 22587778
mexT	mexT	fabH2	fabH2	-	27242034*, 18974177*, 22587778
mexT	mexT	hcnA	hcnA	-	27242034*, 18974177*, 22587778
mexT	mexT	hcnB	hcnB	-	27242034*, 18974177*, 22587778
mexT	mexT	hcnC	hcnC	-	27242034*, 18974177*, 22587778
mexT	mexT	lasB	lasB	-	27242034*, 18974177*, 22587778
mexT	mexT	lldD	lldD	-	27242034*, 18974177*, 22587778
mexT	mexT	lldP	lldP	-	27242034*, 18974177*, 22587778
mexT	mexT	mexE	mexE	+	27242034*, 18974177*, 22587778
mexT	mexT	mexF	mexF	+	27242034*, 18974177*, 22587778
mexT	mexT	mexT	mexT	+	27242034*, 18974177*, 22587778
mexT	mexT	oprD	oprD	-	27242034*, 18974177*, 22587778
mexT	mexT	oprN	oprN	+	27242034*, 18974177*, 22587778
mexT	mexT	PA4851_18680	PA1657	-	27242034*, 18974177*, 22587778
mexT	mexT	PA4851_18675	PA1658	-	27242034*, 18974177*, 22587778
mexT	mexT	PA4851_18245	PA1744	+	27242034*, 18974177*, 22587778

Regulatory gene	Ortholog of the regulatory gene	Target gene	Ortholog of the target gene	Mode of regulation	Reference (PubMed)
mexT	mexT	PA4851_17590	PA1869	-	27242034*, 18974177*, 22587778
mexT	mexT	PA4851_17100	PA1970	+	27242034*, 18974177*, 22587778
mexT	mexT	PA4851_14230	PA2486	+	27242034*, 18974177*, 22587778
mexT	mexT	PA4851_11255	PA2759	+	27242034*, 18974177*, 22587778
mexT	mexT	PA4851_10765	PA2811	+	27242034*, 18974177*, 22587778
mexT	mexT	PA4851_10760	PA2812	+	27242034*, 18974177*, 22587778
mexT	mexT	PA4851_10755	PA2813	+	27242034*, 18974177*, 22587778
mexT	mexT	PA4851_08740	PA3205	+	27242034*, 18974177*, 22587778
mexT	mexT	PA4851_08620	PA3229	+	27242034*, 18974177*, 22587778
mexT	mexT	PA4851_08120	PA3326	-	27242034*, 18974177*, 22587778
mexT	mexT	PA4851_08095	PA3331	-	27242034*, 18974177*, 22587778
mexT	mexT	PA4851_08090	PA3332	-	27242034*, 18974177*, 22587778
mexT	mexT	PA4851_04135	PA4141	+	27242034*, 18974177*, 22587778
mexT	mexT	PA4851_24650	PA4354	+	27242034*, 18974177*, 22587778
mexT	mexT	PA4851_24655	PA4355	+	27242034*, 18974177*, 22587778
mexT	mexT	PA4851_26625	PA4623	+	27242034*, 18974177*, 22587778
mexT	mexT	PA4851_27455	PA4772	-	27242034*, 18974177*, 22587778
mexT	mexT	PA4851_28000	PA4881	+	27242034*, 18974177*, 22587778
mexT	mexT	phnA	phnA	-	27242034*, 18974177*, 22587778
mexT	mexT	phnB	phnB	-	27242034*, 18974177*, 22587778
mexT	mexT	pqsA	pqsA	-	27242034*, 18974177*, 22587778
mexT	mexT	pqsB	pqsB	-	27242034*, 18974177*, 22587778
mexT	mexT	pqsC	pqsC	-	27242034*, 18974177*, 22587778
mexT	mexT	pqsD	pqsD	-	27242034*, 18974177*, 22587778
mexT	mexT	pqsE	pqsE	-	27242034*, 18974177*, 22587778
mexT	mexT	pscE	pscE	-	27242034*, 18974177*, 22587778
mexT	mexT	pvdA	pvdA	+	27242034*, 18974177*, 22587778
mexT	mexT	PA4851_14205	qrh	+	27242034*, 18974177*, 22587778
mexT	mexT	rhlA	rhlA	-	27242034*, 18974177*, 22587778
mexT	mexT	rhlI	rhlI	+	27242034*, 18974177*, 22587778
mexT	mexT	xenB	xenB	+	27242034*, 18974177*, 22587778
mmsR	mmsR	mmsB	mmsB	?	27242034*
mmsR	mmsR	mmsA	mmsA	?	27242034*
mucA	mucA	algU	algU	-	27242034*, 18974177*, 22587778
mucB	mucB	algU	algU	-	27242034*, 18974177*, 22587778
mucC	mucC	algU	algU	-	27242034*, 18974177*, 22587778
mucD	mucD	algU	algU	-	27242034*, 18974177*, 22587778
mvfR	mvfR	mvfR	mvfR	-	27242034*, 18974177*, 22587778
mvfR	mvfR	phnA	phnA	+	27242034*, 18974177*, 22587778
mvfR	mvfR	phnB	phnB	+	27242034*, 18974177*, 22587778
mvfR	mvfR	pqsA	pqsA	+	27242034*, 18974177*, 22587778
mvfR	mvfR	pqsB	pqsB	+	27242034*, 18974177*, 22587778
mvfR	mvfR	pqsC	pqsC	+	27242034*, 18974177*, 22587778
mvfR	mvfR	pqsD	pqsD	+	27242034*, 18974177*, 22587778
mvfR	mvfR	pqsE	pqsE	+	27242034*, 18974177*, 22587778

Regulatory gene	Ortholog of the regulatory gene	Target gene	Ortholog of the target gene	Mode of regulation	Reference (PubMed)
mvfR	mvfR	rhlI	rhlI	+	27242034*, 18974177*, 22587778
mvfR	mvfR	rsmA	rsmA	?	27242034*
mvfR	mvfR	mexH	mexH	?	27242034*
mvfR	mvfR	opmD	opmD	?	27242034*
mvfR	mvfR	mexI	mexI	?	27242034*
mvfR	mvfR	mexG	mexG	?	27242034*
nalC	nalC	mexA	mexA	-	27242034*, 18974177*, 22587778
nalC	nalC	mexB	mexB	-	27242034*, 18974177*, 22587778
nalC	nalC	oprM	oprM	-	27242034*, 18974177*, 22587778
nalD	nalD	mexB	mexB	?	27242034*
nalD	nalD	oprM	oprM	?	27242034*
nalD	nalD	mexA	mexA	?	27242034*
narL	narL	hemA	hemA	d	27242034*, 18974177*, 22587778
narL	narL	hemK	hemK	d	27242034*, 18974177*, 22587778
narL	narL	moeB	moeB	d	27242034*, 18974177*, 22587778
narL	narL	murI	murI	d	27242034*, 18974177*, 22587778
narL	narL	narH	narH	+	27242034*, 18974177*, 22587778
narL	narL	narI	narI	+	27242034*, 18974177*, 22587778
narL	narL	narJ	narJ	+	27242034*, 18974177*, 22587778
narL	narL	narK1	narK1	+	27242034*, 18974177*, 22587778
narL	narL	narK2	narK2	+	27242034*, 18974177*, 22587778
narL	narL	nirQ	nirQ	+	27242034*, 18974177*, 22587778
narL	narL	prfA	prfA	d	27242034*, 18974177*, 22587778
narL	narL	narG	narG	?	27242034*, 18974177*, 22587778
narL	narL	arcD	arcD	?	27242034*, 18974177*, 22587778
narL	narL	arcC	arcC	?	27242034*, 18974177*, 22587778
narL	narL	arcB	arcB	?	27242034*, 18974177*, 22587778
narL	narL	arcA	arcA	?	27242034*, 18974177*, 22587778
nfxB	nfxB	oprJ	oprJ	?	27242034*
nfxB	nfxB	mexD	mexD	?	27242034*
nfxB	nfxB	mexC	mexC	?	27242034*
nfxB	nfxB	nfxB	nfxB	-	27242034*
np20	np20	np20	np20	-	27242034*
np20	np20	znuC	znuC	-	27242034*
np20	np20	znuB	znuB	-	27242034*
np20	np20	PA4851_23690	PA0781	-	27242034*
np20	np20	PA4851_06830	PA3601	-	27242034*
np20	np20	PA4851_06835	PA3600	-	27242034*
np20	np20	PA4851_31375	PA5536	-	27242034*
np20	np20	PA4851_31370	PA5535	-	27242034*
np20	np20	PA4851_31365	PA5534	-	27242034*
np20	np20	PA4851_27785	PA4838	-	27242034*
np20	np20	PA4851_31380	PA5537	-	27242034*
np20	np20	PA4851_31390	PA5539	-	27242034*
np20	np20	PA4851_31395	PA5540	-	27242034*

Regulatory gene	Ortholog of the regulatory gene	Target gene	Ortholog of the target gene	Mode of regulation	Reference (PubMed)
np20	np20	pyrQ	pyrQ	-	27242034*
np20	np20	amiA	amiA	-	27242034*
np20	np20	PA4851_04525	PA4063	-	27242034*
np20	np20	PA4851_31185	PA5498	-	27242034*
NrdR	NrdR	nrdD	nrdD	-	27242034*
NrdR	NrdR	nrdG	nrdG	-	27242034*
NrdR	NrdR	nrdJb	nrdJb	-	27242034*
NrdR	NrdR	nrdJa	nrdJa	-	27242034*
NrdR	NrdR	nrdA	nrdA	-	27242034*
NrdR	NrdR	nrdB	nrdB	-	27242034*
NrdR	NrdR	topA	topA	-	27242034*
NtrC	NtrC	ntrB	ntrB	+	27242034*, 18974177*, 22587778
NtrC	NtrC	ntrC	ntrC	+	27242034*, 18974177*, 22587778
NtrC	NtrC	PA4851_18315	PA1730	+	27242034*, 18974177*, 22587778
NtrC	NtrC	PA4851_18310	PA1731	+	27242034*, 18974177*, 22587778
NtrC	NtrC	PA4851_18305	PA1732	+	27242034*, 18974177*, 22587778
NtrC	NtrC	glnA	glnA	+	27242034*, 18974177*, 22587778
NtrC	NtrC	glnK	glnK	+	27242034*, 18974177*, 22587778
NtrC	NtrC	amtB	amtB	+	27242034*, 18974177*, 22587778
ospR	ospR	PA4851_10675	PA2826	-	27242034*
oxyR	oxyR	katB	katB	?	27242034*
oxyR	oxyR	PA4851_23355	PA0848	?	27242034*
oxyR	oxyR	PA4851_26565	PA4612	?	27242034*
PA4851_00625	PA0120	dctA	dctA	-	27242034*
PA4851_00625	PA0120	PA4851_00625	PA0120	-	27242034*
PA4851_00860	PA0167	PA4851_19395	PA1517	-	27242034*, 18974177*, 22587778
PA4851_00860	PA0167	PA4851_19400	PA1516	-	27242034*, 18974177*, 22587778
PA4851_00860	PA0167	alc	alc	-	27242034*, 18974177*, 22587778
PA4851_00860	PA0167	PA4851_19410	PA1514	-	27242034*, 18974177*, 22587778
PA4851_00860	PA0167	PA4851_19415	PA1513	-	27242034*, 18974177*, 22587778
PA4851_00860	PA0167	PA4851_00705	PA0136	-	27242034*, 18974177*, 22587778
PA4851_00860	PA0167	PA4851_00710	PA0137	-	27242034*, 18974177*, 22587778
PA4851_00860	PA0167	PA4851_00715	PA0138	-	27242034*, 18974177*, 22587778
PA4851_00860	PA0167	PA4851_00850	PA0165	-	27242034*, 18974177*, 22587778
PA4851_00860	PA0167	PA4851_00855	PA0166	-	27242034*, 18974177*, 22587778
PA4851_00860	PA0167	PA4851_00860	PA0167	-	27242034*, 18974177*, 22587778
PA4851_00860	PA0167	PA4851_00865	PA0168	-	27242034*, 18974177*, 22587778
PA4851_01350	PA0268	PA4851_01355	PA0269	-	27242034*
PA4851_01350	PA0268	PA4851_01360	PA0270	-	27242034*
PA4851_02195	PA0436	PA4851_02230	PA0443	-	27242034*
PA4851_02195	PA0436	PA4851_02235	PA0444	-	27242034*
PA4851_02195	PA0436	dht	dht	-	27242034*
PA4851_02195	PA0436	PA4851_02215	PA0440	-	27242034*
PA4851_02195	PA0436	PA4851_02210	PA0439	-	27242034*
PA4851_02195	PA0436	codB	codB	-	27242034*

Regulatory gene	Ortholog of the regulatory gene	Target gene	Ortholog of the target gene	Mode of regulation	Reference (PubMed)
PA4851_02195	PA0436	codA	codA	-	27242034*
PA4851_02445	modE	modA	modA	-	27242034*
PA4851_02445	modE	modB	modB	-	27242034*
PA4851_02445	modE	modC	modC	-	27242034*
PA4851_02745	PA0547	PA4851_02745	PA0547	-	27242034*, 18974177*, 22587778
PA4851_02745	PA0547	metK	metK	-	27242034*, 18974177*, 22587778
PA4851_04015	PA4165	PA4851_04010	PA4166	-	27242034*
PA4851_04180	PA4132	cysI	cysI	-	27242034*
PA4851_04180	PA4132	PA4851_04195	PA4129	-	27242034*
PA4851_04180	PA4132	PA4851_04175	PA4133	-	27242034*
PA4851_06045	PA3757	PA4851_06045	PA3757	-	27242034*
PA4851_06045	PA3757	PA4851_06040	PA3758	-	27242034*
PA4851_06045	PA3757	PA4851_06035	PA3759	-	27242034*
PA4851_06045	PA3757	PA4851_06030	PA3760	-	27242034*
PA4851_06045	PA3757	PA4851_06025	PA3761	-	27242034*
PA4851_06345	PA3697	fhp	fhp	+	27242034*, 18974177*, 22587778
PA4851_06385	PA3689	PA4851_06380	PA3690	+	27242034*
PA4851_07825	PA3381	PA4851_07825	PA3381	-	27242034*
PA4851_07825	PA3381	PA4851_07830	PA3380	-	27242034*
PA4851_07825	PA3381	PA4851_07835	PA3379	-	27242034*
PA4851_07825	PA3381	PA4851_07840	PA3378	-	27242034*
PA4851_07825	PA3381	PA4851_07845	PA3377	-	27242034*
PA4851_07825	PA3381	PA4851_07850	PA3376	-	27242034*
PA4851_07825	PA3381	PA4851_07855	PA3375	-	27242034*
PA4851_07825	PA3381	PA4851_07860	PA3374	-	27242034*
PA4851_07825	PA3381	PA4851_07865	PA3373	-	27242034*
PA4851_07825	PA3381	PA4851_07870	PA3372	-	27242034*
PA4851_08520	PA3249	PA4851_08520	PA3249	-	27242034*
PA4851_08520	PA3249	PA4851_08515	PA3250	-	27242034*
PA4851_08520	PA3249	PA4851_08510	PA3251	-	27242034*
PA4851_08520	PA3249	PA4851_08505	PA3252	-	27242034*
PA4851_08520	PA3249	PA4851_08500	PA3253	-	27242034*
PA4851_08520	PA3249	PA4851_08495	PA3254	-	27242034*
PA4851_08520	PA3249	PA4851_08490	PA3255	-	27242034*
PA4851_08520	PA3249	PA4851_10810	PA2802	-	27242034*
PA4851_08520	PA3249	PA4851_10805	PA2803	-	27242034*
PA4851_08520	PA3249	PA4851_10800	PA2804	-	27242034*
PA4851_08520	PA3249	PA4851_19125	PA1144	-	27242034*
PA4851_08520	PA3249	PA4851_21585	PA1143	-	27242034*
PA4851_08520	PA3249	PA4851_21590	PA1142	-	27242034*
PA4851_08845	PA3184	PA4851_08845	PA3184	-	27242034*
PA4851_08845	PA3184	edd	edd	-	27242034*
PA4851_08845	PA3184	glk	glk	-	27242034*
PA4851_08845	PA3184	gltR	gltR	-	27242034*
PA4851_08845	PA3184	gltS	gltS	-	27242034*

Regulatory gene	Ortholog of the regulatory gene	Target gene	Ortholog of the target gene	Mode of regulation	Reference (PubMed)
PA4851_08845	PA3184	gapA	gapA	-	27242034*
PA4851_08845	PA3184	zwf	zwf	-	27242034*
PA4851_08845	PA3184	pgl	pgl	-	27242034*
PA4851_08845	PA3184	PA4851_08860	PA3181	-	27242034*
PA4851_11865	fhpR	fhp	fhp	+	27242034*, 18974177*, 22587778
PA4851_11865	fhpR	PA4851_11865	fhpR	-	27242034*, 18974177*, 22587778
PA4851_11865	fhpR	ppyR	ppyR	+	27242034*, 18974177*, 22587778
PA4851_11865	fhpR	PA4851_11880	PA2662	+	27242034*, 18974177*, 22587778
PA4851_11910	bqsR/PA2657	phnA	phnA	+	27242034*, 18974177*, 22587778
PA4851_11910	bqsR/PA2657	pqsA	pqsA	+	27242034*, 18974177*, 22587778
PA4851_11910	bqsR/PA2657	rhlA	rhlA	+	27242034*, 18974177*, 22587778
PA4851_11910	bqsR/PA2657	rhlB	rhlB	+	27242034*, 18974177*, 22587778
PA4851_11915	bqsS/PA2656	phnA	phnA	+	27242034*, 18974177*, 22587778
PA4851_11915	bqsS/PA2656	pqsA	pqsA	+	27242034*, 18974177*, 22587778
PA4851_11915	bqsS/PA2656	rhlA	rhlA	+	27242034*, 18974177*, 22587778
PA4851_11915	bqsS/PA2656	rhlB	rhlB	+	27242034*, 18974177*, 22587778
PA4851_12250	PA2591	pprB	pprB	-	27242034*, 18974177*, 22587778
PA4851_14400	PA2449	gcvH2	gcvH2	+	27242034*
PA4851_14400	PA2449	gcvP2	gcvP2	+	27242034*
PA4851_14400	PA2449	glyA2	glyA2	+	27242034*
PA4851_14400	PA2449	sdaA	sdaA	+	27242034*
PA4851_14400	PA2449	gcvT2	gcvT2	+	27242034*
PA4851_15145	PA2299	PA4851_15145	PA2299	-	27242034*
PA4851_15145	PA2299	PA4851_15150	PA2298	-	27242034*
PA4851_15145	PA2299	PA4851_15155	PA2297	-	27242034*
PA4851_15145	PA2299	PA4851_15160	PA2296	-	27242034*
PA4851_15145	PA2299	PA4851_15165	PA2295	-	27242034*
PA4851_15145	PA2299	PA4851_15170	PA2294	-	27242034*
PA4851_15145	PA2299	PA4851_15175	PA2293	-	27242034*
PA4851_15145	PA2299	PA4851_15180	PA2292	-	27242034*
PA4851_16790	PA2032	PA4851_16795	PA2031	-	27242034*
PA4851_16900	PA2010	PA4851_16900	PA2010	-	27242034*
PA4851_16900	PA2010	hmgA	hmgA	-	27242034*
PA4851_16900	PA2010	fahA	fahA	-	27242034*
PA4851_16900	PA2010	maiA	maiA	-	27242034*
PA4851_16900	PA2010	PA4851_16920	PA2006	-	27242034*
PA4851_19280	PA1539	PA4851_19280	PA1539	-	27242034*
PA4851_19280	PA1539	PA4851_19285	PA1538	-	27242034*
PA4851_19280	PA1539	PA4851_19290	PA1537	-	27242034*
PA4851_19380	PA1520	PA4851_10110	PA2938	-	27242034*, 18974177*, 22587778
PA4851_19380	PA1520	PA4851_19380	PA1520	-	27242034*, 18974177*, 22587778
PA4851_19380	PA1520	PA4851_19390	PA1518	-	27242034*, 18974177*, 22587778
PA4851_19380	PA1520	gcl	gcl	-	27242034*, 18974177*, 22587778
PA4851_19380	PA1520	PA4851_19475	PA1501	-	27242034*, 18974177*, 22587778
PA4851_19380	PA1520	PA4851_19480	PA1500	-	27242034*, 18974177*, 22587778

Regulatory gene	Ortholog of the regulatory gene	Target gene	Ortholog of the target gene	Mode of regulation	Reference (PubMed)
PA4851_19380	PA1520	PA4851_00850	PA0165	-	27242034*, 18974177*, 22587778
PA4851_19380	PA1520	PA4851_19465	PA1503	-	27242034*, 18974177*, 22587778
PA4851_19380	PA1520	PA4851_19445	PA1507	-	27242034*, 18974177*, 22587778
PA4851_19380	PA1520	PA4851_19395	PA1517	-	27242034*, 18974177*, 22587778
PA4851_19380	PA1520	PA4851_19400	PA1516	-	27242034*, 18974177*, 22587778
PA4851_19380	PA1520	PA4851_19410	PA1514	-	27242034*, 18974177*, 22587778
PA4851_19380	PA1520	PA4851_19415	PA1513	-	27242034*, 18974177*, 22587778
PA4851_19380	PA1520	PA4851_02390	PA0476	-	27242034*, 18974177*, 22587778
PA4851_19380	PA1520	PA4851_19385	PA1519	-	27242034*, 18974177*, 22587778
PA4851_19380	PA1520	alc	alc	-	27242034*, 18974177*, 22587778
PA4851_19460	PA1504	xdhA	xdhA	-	27242034*, 18974177*, 22587778
PA4851_19460	PA1504	xdhB	xdhB	-	27242034*, 18974177*, 22587778
PA4851_19460	PA1504	PA4851_19370	PA1522	-	27242034*, 18974177*, 22587778
PA4851_19460	PA1504	PA4851_19375	PA1521	-	27242034*, 18974177*, 22587778
PA4851_19460	PA1504	PA4851_19385	PA1519	-	27242034*, 18974177*, 22587778
PA4851_20955	PA1269	PA4851_20955	PA1269	+	27242034*
PA4851_20955	PA1269	PA4851_20960	PA1268	+	27242034*
PA4851_20955	PA1269	PA4851_20965	PA1267	+	27242034*
PA4851_20955	PA1269	PA4851_21000	PA1260	+	27242034*
PA4851_20955	PA1269	PA4851_21005	PA1259	+	27242034*
PA4851_20955	PA1269	PA4851_21010	PA1258	+	27242034*
PA4851_20955	PA1269	PA4851_21015	PA1257	+	27242034*
PA4851_20955	PA1269	PA4851_21020	PA1256	+	27242034*
PA4851_20955	PA1269	PA4851_21025	PA1255	+	27242034*
PA4851_20955	PA1269	PA4851_21030	PA1254	+	27242034*
PA4851_20955	PA1269	PA4851_21035	PA1253	+	27242034*
PA4851_22055	PA1050	PA4851_22050	PA1051	+	27242034*
PA4851_22055	PA1050	PA4851_22045	PA1052	+	27242034*
PA4851_22615	pqrR	PA4851_22620	PA0941	-	27242034*
PA4851_22615	pqrR	PA4851_22625	PA0940	-	27242034*
PA4851_22615	pqrR	PA4851_22630	PA0939	-	27242034*
PA4851_22615	pqrR	PA4851_22615	pqrR	+	27242034*
PA4851_23610	PA0797	PA4851_23610	PA0797	-	27242034*
PA4851_23610	PA0797	prpB	prpB	-	27242034*
PA4851_23610	PA0797	prpC	prpC	-	27242034*
PA4851_23610	PA0797	PA4851_23625	PA0794	-	27242034*
PA4851_23610	PA0797	PA4851_23630	PA0793	-	27242034*
PA4851_23610	PA0797	prpD	prpD	-	27242034*
PA4851_23700	PA0779	fhp	fhp	+	27242034*, 18974177*, 22587778
PA4851_23700	PA0779	PA4851_11865	fhpR	-	27242034*, 18974177*, 22587778
PA4851_24455	mvaT	cupA1	cupA1	-	27242034*, 18974177*, 22587778
PA4851_24455	mvaT	cupB1	cupB1	-	27242034*, 18974177*, 22587778
PA4851_24455	mvaT	cupC1	cupC1	-	27242034*, 18974177*, 22587778
PA4851_24455	mvaT	ptxS	ptxS	+	27242034*, 18974177*, 22587778
PA4851_25390	psdR	PA4851_25395	PA4500	-	27242034*

Regulatory gene	Ortholog of the regulatory gene	Target gene	Ortholog of the target gene	Mode of regulation	Reference (PubMed)
PA4851_25390	psdR	PA4851_25410	dppB	-	27242034*
PA4851_25390	psdR	PA4851_25415	dppC	-	27242034*
PA4851_25390	psdR	PA4851_25420	dppD	-	27242034*
PA4851_25390	psdR	PA4851_25425	dppF	-	27242034*
PA4851_26805	PA4659	PA4851_26795	PA4657	-	27242034*
PA4851_26805	PA4659	PA4851_26800	PA4658	-	27242034*
PA4851_26805	PA4659	PA4851_26805	PA4659	-	27242034*
PA4851_26805	PA4659	phr	phr	-	27242034*
PA4851_27440	PA4769	PA4851_27440	PA4769	-	27242034*, 18974177*, 22587778
PA4851_27440	PA4769	lldP	lldP	-	27242034*, 18974177*, 22587778
PA4851_27440	PA4769	lldD	lldD	-	27242034*, 18974177*, 22587778
PA4851_27440	PA4769	PA4851_27455	PA4772	-	27242034*, 18974177*, 22587778
PA4851_28125	PA4906	vanA	vanA	-	27242034*
PA4851_28125	PA4906	vanB	vanB	-	27242034*
PA4851_28125	PA4906	PA4851_28110	PA4903	-	27242034*
PA4851_28175	PA4916	nadD	nadD	-	27242034*, 18974177*, 22587778
PA4851_28175	PA4916	PA4851_28175	PA4916	-	27242034*, 18974177*, 22587778
PA4851_28175	PA4916	PA4851_28185	PA4918	-	27242034*, 18974177*, 22587778
PA4851_28175	PA4916	pncB1	pncB1	-	27242034*, 18974177*, 22587778
PA4851_28175	PA4916	nadE	nadE	-	27242034*, 18974177*, 22587778
PA4851_30850	PA5431	PA4851_30855	PA5432	-	27242034*
PA4851_30850	PA5431	PA4851_30860	PA5433	-	27242034*
PA4851_30880	PSPA7_RS29685	PA4851_30870	oadA	?	27242034*, 18974177*, 22587778
PA4851_30880	PSPA7_RS29685	PA4851_00880	PSPA7_RS01190	?	27242034*, 18974177*, 22587778
PA4851_30880	PSPA7_RS29685	norC	norC	?	27242034*, 18974177*, 22587778
PA4851_30880	PSPA7_RS29685	PA4851_30880	PSPA7_RS29685	?	27242034*, 18974177*, 22587778
PA4851_30880	PSPA7_RS29685	nosZ	nosZ	?	27242034*, 18974177*, 22587778
PA4851_30885	PA5438	zwf	zwf	-	27242034*, 18974177*, 22587778
PA4851_30885	PA5438	aceE	aceE	-	27242034*, 18974177*, 22587778
PA4851_30885	PA5438	aceF	aceF	-	27242034*, 18974177*, 22587778
PA4851_30885	PA5438	PA4851_30885	PA5438	-	27242034*, 18974177*, 22587778
PA4851_30885	PA5438	PA4851_08840	PA3185	-	27242034*, 18974177*, 22587778
PA4851_31225	PA5506	PA4851_31225	PA5506	-	27242034*
PA4851_31225	PA5506	PA4851_31230	PA5507	-	27242034*
PA4851_31225	PA5506	PA4851_31235	PA5508	-	27242034*
PA4851_31225	PA5506	PA4851_31240	PA5509	-	27242034*
PA4851_31225	PA5506	PA4851_31245	PA5510	-	27242034*
pchR	pchR	fptA	fptA	+	27242034*, 18974177*, 22587778
pchR	pchR	pchA	pchA	+	27242034*, 18974177*, 22587778
pchR	pchR	pchB	pchB	+	27242034*, 18974177*, 22587778
pchR	pchR	pchC	pchC	+	27242034*, 18974177*, 22587778
pchR	pchR	pchD	pchD	+	27242034*, 18974177*, 22587778
pchR	pchR	pchE	pchE	+	27242034*, 18974177*, 22587778
pchR	pchR	pchF	pchF	+	27242034*, 18974177*, 22587778
pchR	pchR	pchR	pchR	-	27242034*, 18974177*, 22587778

Regulatory gene	Ortholog of the regulatory gene	Target gene	Ortholog of the target gene	Mode of regulation	Reference (PubMed)
pepA	pepA	algD	algD	?	27242034*, 18974177*, 22587778
pfeR	pfeR	pfeA	pfeA	+	27242034*, 18974177*, 22587778
phhR	phhR	phhA	phhA	+	27242034*
phhR	phhR	phhB	phhB	+	27242034*
phhR	phhR	phhC	phhC	+	27242034*
phhR	phhR	phhR	phhR	-	27242034*
phhR	phhR	hpd	hpd	+	27242034*
phhR	phhR	phhR	phhR	+	27242034*
phhR	phhR	phhA	phhA	+	27242034*
phhR	phhR	phhB	phhB	+	27242034*
phhR	phhR	phhC	phhC	+	27242034*
phoB	phoB	PA4851_27815	PSPA7_RS26570 (PSPA7_5564)	?	27242034*
phoB	phoB	phoU	phoU	?	27242034*
phoP	phoP	PA4851_23420	PA0836	-	27242034*, 18974177*, 22587778
phoP	phoP	gabD	gabD	+	27242034*, 18974177*, 22587778
phoP	phoP	gabT	gabT	+	27242034*, 18974177*, 22587778
phoP	phoP	PA4851_07225	PA3522	+	27242034*, 18974177*, 22587778
phoP	phoP	oprH	oprH	+	27242034*, 18974177*, 22587778
phoP	phoP	PA4851_22725	PA0921	+	27242034*, 18974177*, 22587778
phoP	phoP	PA4851_21320	PA1196	-	27242034*, 18974177*, 22587778
phoP	phoP	PA4851_08205	PA3309	-	27242034*, 18974177*, 22587778
phoP	phoP	PA4851_06585	PA3649	+	27242034*, 18974177*, 22587778
phoP	phoP	PA4851_04785	PA4010	+	27242034*, 18974177*, 22587778
phoP	phoP	PA4851_04780	PA4011	+	27242034*, 18974177*, 22587778
phoP	phoP	PA4851_25160	PA4453	+	27242034*, 18974177*, 22587778
phoP	phoP	PA4851_25165	PA4454	+	27242034*, 18974177*, 22587778
phoP	phoP	PA4851_25170	PA4455	+	27242034*, 18974177*, 22587778
phoP	phoP	PA4851_28185	PA4918	-	27242034*, 18974177*, 22587778
phoP	phoP	phoP	phoP	+	27242034*, 18974177*, 22587778
phoP	phoP	phoQ	phoQ	+	27242034*, 18974177*, 22587778
phoP	phoP	sodB	sodB	+	27242034*, 18974177*, 22587778
phoP	phoP	tpbA	tpbA	+	27242034*, 18974177*, 22587778
phoP	phoP	ackA	ackA	-	27242034*
phoP	phoP	arnT	arnT	?	27242034*
phoP	phoP	arnC	arnC	?	27242034*
phoP	phoP	arnB	arnB	?	27242034*
phoP	phoP	arnA	arnA	?	27242034*
phoP	phoP	arnF	arnF	?	27242034*
phoP	phoP	arnE	arnE	?	27242034*
phoP	phoP	arnD	arnD	?	27242034*
phoP	phoP	PA4851_07040	PA3559	?	27242034*
phoP	phoP	PA4851_20580	PA1343	?	27242034*
phoQ	phoQ	algR	algR	-	27242034*, 18974177*, 22587778
phoQ	phoQ	arnB	arnB	-	27242034*, 18974177*, 22587778
phoQ	phoQ	pmrA	pmrA	-	27242034*, 18974177*, 22587778

Regulatory gene	Ortholog of the regulatory gene	Target gene	Ortholog of the target gene	Mode of regulation	Reference (PubMed)
pilR	pilR	pilA	pilA	-	27242034*
pilR	pilR	pilR	pilR	+	27242034*
pilR	pilR	pilS	pilS	+	27242034*
pmrA	pmrA	cysT	cysT	+	27242034*, 18974177*, 22587778
pmrA	pmrA	dnr	dnr	-	27242034*, 18974177*, 22587778
pmrA	pmrA	PA4851_24675	feoA	+	27242034*, 18974177*, 22587778
pmrA	pmrA	PA4851_24670	feoB	+	27242034*, 18974177*, 22587778
pmrA	pmrA	metK	metK	+	27242034*, 18974177*, 22587778
pmrA	pmrA	mexG	mexG	-	27242034*, 18974177*, 22587778
pmrA	pmrA	mexH	mexH	-	27242034*, 18974177*, 22587778
pmrA	pmrA	mexI	mexI	-	27242034*, 18974177*, 22587778
pmrA	pmrA	mgtA	mgtA	+	27242034*, 18974177*, 22587778
pmrA	pmrA	mgtE	mgtE	+	27242034*, 18974177*, 22587778
pmrA	pmrA	PA4851_01035	PA0201	+	27242034*, 18974177*, 22587778
pmrA	pmrA	PA4851_14850	PA2359	+	27242034*, 18974177*, 22587778
pmrA	pmrA	PA4851_07500	PA3446	+	27242034*, 18974177*, 22587778
pmrA	pmrA	PA4851_07260	PA3515	-	27242034*, 18974177*, 22587778
pmrA	pmrA	PA4851_07255	PA3516	-	27242034*, 18974177*, 22587778
pmrA	pmrA	PA4851_07250	PA3517	-	27242034*, 18974177*, 22587778
pmrA	pmrA	PA4851_07245	PA3518	-	27242034*, 18974177*, 22587778
pmrA	pmrA	PA4851_27460	PA4773	+	27242034*, 18974177*, 22587778
pmrA	pmrA	PA4851_27465	PA4774	+	27242034*, 18974177*, 22587778
pmrA	pmrA	PA4851_27470	PA4775	+	27242034*, 18974177*, 22587778
pmrA	pmrA	PA4851_27500	PA4781	+	27242034*, 18974177*, 22587778
pmrA	pmrA	PA4851_27505	PA4782	+	27242034*, 18974177*, 22587778
pmrA	pmrA	PA4851_27705	PA4822	+	27242034*, 18974177*, 22587778
pmrA	pmrA	PA4851_27710	PA4823	+	27242034*, 18974177*, 22587778
pmrA	pmrA	PA4851_27715	PA4824	+	27242034*, 18974177*, 22587778
pmrA	pmrA	PA4851_27725	PA4826	+	27242034*, 18974177*, 22587778
pmrA	pmrA	pcoA	pcoA	-	27242034*, 18974177*, 22587778
pmrA	pmrA	pcoB	pcoB	-	27242034*, 18974177*, 22587778
pmrA	pmrA	pmrA	pmrA	+	27242034*, 18974177*, 22587778
pmrA	pmrA	pmrB	pmrB	+	27242034*, 18974177*, 22587778
pmrA	pmrA	PA4851_24590	PA0202	+	27242034*, 18974177*, 22587778
pmrA	pmrA	PA4851_19170	PA1559	+	27242034*, 18974177*, 22587778
pmrA	pmrA	cueR	cueR	+	27242034*, 18974177*, 22587778
pmrA	pmrA	arnE	arnE	?	27242034*
pmrA	pmrA	arnD	arnD	?	27242034*
pmrA	pmrA	arnT	arnT	?	27242034*
pmrA	pmrA	arnC	arnC	?	27242034*
pmrA	pmrA	arnB	arnB	?	27242034*
pmrA	pmrA	arnA	arnA	?	27242034*
pmrA	pmrA	arnF	arnF	?	27242034*
pmrA	pmrA	PA4851_07040	PA3559	?	27242034*
pprB	pprB	rpoS	rpoS	+	27242034*, 18974177*, 22587778

Regulatory gene	Ortholog of the regulatory gene	Target gene	Ortholog of the target gene	Mode of regulation	Reference (PubMed)
pprB	pprB	PA4851_12250	PA2591	+	27242034*, 18974177*, 22587778
pprB	pprB	rsaL	rsaL	?	27242034*
pprB	pprB	tadZ	tadZ	?	27242034*
pprB	pprB	flp	flp	?	27242034*
pprB	pprB	rcpC	rcpC	?	27242034*
pprB	pprB	fppA	fppA	?	27242034*
pprB	pprB	pprB	pprB	?	27242034*
pprB	pprB	rcpA	rcpA	?	27242034*
pprB	pprB	lasB	lasB	?	27242034*
pprB	pprB	pprA	pprA	?	27242034*
pprB	pprB	PA4851_24350	PA4294	?	27242034*
pprB	pprB	lasI	lasI	?	27242034*
pprB	pprB	PA4851_24370	PA4298	?	27242034*
pprB	pprB	tadA	tadA	?	27242034*
pprB	pprB	tadG	tadG	?	27242034*
pprB	pprB	tadC	tadC	?	27242034*
pprB	pprB	tadB	tadB	?	27242034*
ppyR	ppyR	lasB	lasB	-	27242034*, 18974177*, 22587778
psrA	psrA	etfA	etfA	-	27242034*, 18974177*, 22587778
psrA	psrA	etfB	etfB	-	27242034*, 18974177*, 22587778
psrA	psrA	exoS	exoS	+	27242034*, 18974177*, 22587778
psrA	psrA	exsA	exsA	+	27242034*, 18974177*, 22587778
psrA	psrA	exsB	exsB	+	27242034*, 18974177*, 22587778
psrA	psrA	exsC	exsC	+	27242034*, 18974177*, 22587778
psrA	psrA	exsE	exsE	+	27242034*, 18974177*, 22587778
psrA	psrA	PA4851_18280	fadB	+	27242034*, 18974177*, 22587778
psrA	psrA	mmsR	mmsR	+	27242034*, 18974177*, 22587778
psrA	psrA	PA4851_02535	PA0506	-	27242034*, 18974177*, 22587778
psrA	psrA	PA4851_10030	PA2953	-	27242034*, 18974177*, 22587778
psrA	psrA	PA4851_06860	PA3595	?	27242034*, 18974177*, 22587778
psrA	psrA	psrA	psrA	-	27242034*, 18974177*, 22587778
psrA	psrA	rpoS	rpoS	+	27242034*, 18974177*, 22587778
psrA	psrA	PA4851_02540	PA0507	-	27242034*, 18974177*, 22587778
psrA	psrA	PA4851_02545	PA0508	-	27242034*, 18974177*, 22587778
psrA	psrA	PA4851_17780	PA1831	-	27242034*, 18974177*, 22587778
psrA	psrA	PA4851_17785	PA1830	-	27242034*, 18974177*, 22587778
psrA	psrA	faoA	faoA	-	27242034*, 18974177*, 22587778
psrA	psrA	algQ	algQ	-	27242034*, 18974177*, 22587778
ptxR	ptxR	pqsA	pqsA	-	27242034*, 18974177*, 22587778
ptxR	ptxR	pqsB	pqsB	-	27242034*, 18974177*, 22587778
ptxR	ptxR	pqsC	pqsC	-	27242034*, 18974177*, 22587778
ptxR	ptxR	pqsD	pqsD	-	27242034*, 18974177*, 22587778
ptxR	ptxR	ptxS	ptxS	+	27242034*, 18974177*, 22587778
ptxR	ptxR	pvcA	pvcA	+	27242034*, 18974177*, 22587778
ptxR	ptxR	pvcB	pvcB	+	27242034*, 18974177*, 22587778

Regulatory gene	Ortholog of the regulatory gene	Target gene	Ortholog of the target gene	Mode of regulation	Reference (PubMed)
ptxR	ptxR	pvcC	pvcC	+	27242034*, 18974177*, 22587778
ptxR	ptxR	pvcD	pvcD	+	27242034*, 18974177*, 22587778
ptxR	ptxR	rhlI	rhlI	-	27242034*, 18974177*, 22587778
ptxR	ptxR	toxA	toxA	+	27242034*, 18974177*, 22587778
ptxS	ptxS	PA4851_15325	PA2263	+	27242034*, 18974177*, 22587778
ptxS	ptxS	PA4851_15335	PA2261	?	27242034*, 18974177*, 22587778
ptxS	ptxS	ptxR	ptxR	-	27242034*, 18974177*, 22587778
ptxS	ptxS	ptxS	ptxS	-	27242034*, 18974177*, 22587778
ptxS	ptxS	PA4851_15320	PA2264	-	27242034*, 18974177*, 22587778
ptxS	ptxS	PA4851_15315	PA2265	-	27242034*, 18974177*, 22587778
ptxS	ptxS	PA4851_15310	PA2266	-	27242034*, 18974177*, 22587778
ptxS	ptxS	PA4851_15330	PA2262	+	27242034*, 18974177*, 22587778
ptxS	ptxS	PA4851_15340	PA2260	+	27242034*, 18974177*, 22587778
pvdS	pvdS	pvdS	pvdS	?	27242034*, 18974177*, 22587778
pvdS	pvdS	cat	cat	+	27242034*, 18974177*, 22587778
pvdS	pvdS	grx	grx	+	27242034*, 18974177*, 22587778
pvdS	pvdS	imm2	imm2	?	27242034*, 18974177*, 22587778
pvdS	pvdS	PA4851_14690	PA2390	+	27242034*, 18974177*, 22587778
pvdS	pvdS	PA4851_14685	opmQ	+	27242034*, 18974177*, 22587778
pvdS	pvdS	PA4851_01740	PA0346	+	27242034*, 18974177*, 22587778
pvdS	pvdS	PA4851_23505	PA0818	+	27242034*, 18974177*, 22587778
pvdS	pvdS	pvdR	PA2389	+	27242034*, 18974177*, 22587778
pvdS	pvdS	PA4851_14675	PA2393	+	27242034*, 18974177*, 22587778
pvdS	pvdS	PA4851_14640	PA2402	+	27242034*, 18974177*, 22587778
pvdS	pvdS	PA4851_14590	PA2411	+	27242034*, 18974177*, 22587778
pvdS	pvdS	PA4851_14585	PA2412	+	27242034*, 18974177*, 22587778
pvdS	pvdS	PA4851_14510	PA2427	+	27242034*, 18974177*, 22587778
pvdS	pvdS	PA4851_13670	PA2531	+	27242034*, 18974177*, 22587778
pvdS	pvdS	PA4851_05855	PA3794	?	27242034*, 18974177*, 22587778
pvdS	pvdS	PA4851_24830	PA4390	?	27242034*, 18974177*, 22587778
pvdS	pvdS	PA4851_27760	PA4833	+	27242034*, 18974177*, 22587778
pvdS	pvdS	PA4851_29245	PA5130	+	27242034*, 18974177*, 22587778
pvdS	pvdS	PA4851_29395	PA5150	+	27242034*, 18974177*, 22587778
pvdS	pvdS	PA4851_29610	PA5190	?	27242034*, 18974177*, 22587778
pvdS	pvdS	piv	prpL	+	27242034*, 18974177*, 22587778
pvdS	pvdS	ptxR	ptxR	+	27242034*, 18974177*, 22587778
pvdS	pvdS	pvcA	pvcA	+	27242034*, 18974177*, 22587778
pvdS	pvdS	pvcB	pvcB	+	27242034*, 18974177*, 22587778
pvdS	pvdS	pvcC	pvcC	+	27242034*, 18974177*, 22587778
pvdS	pvdS	pvcD	pvcD	+	27242034*, 18974177*, 22587778
pvdS	pvdS	pvdA	pvdA	+	27242034*, 18974177*, 22587778
pvdS	pvdS	PA4851_14655	pvdE	+	27242034*, 18974177*, 22587778
pvdS	pvdS	pvdF	pvdF	+	27242034*, 18974177*, 22587778
pvdS	pvdS	pvdG	pvdG	+	27242034*, 18974177*, 22587778
pvdS	pvdS	pvdH	pvdH	+	27242034*, 18974177*, 22587778

Regulatory gene	Ortholog of the regulatory gene	Target gene	Ortholog of the target gene	Mode of regulation	Reference (PubMed)
pvdS	pvdS	PA4851_14670	pvdN	+	27242034*, 18974177*, 22587778
pvdS	pvdS	pys2	pys2	?	27242034*, 18974177*, 22587778
pvdS	pvdS	toxR	toxR	+	27242034*, 18974177*, 22587778
pvdS	pvdS	toxA	toxA	+	27242034*, 18974177*, 22587778
pvdS	pvdS	PA4851_14390	PA2452	+	27242034*, 18974177*, 22587778
pvdS	pvdS	pvdL	pvdL	+	27242034*, 18974177*, 22587778
qscR	qscR	rsaL	rsaL	-	27242034*, 18974177*, 22587778
qscR	qscR	PA4851_17450	PA1897	+	27242034*, 18974177*, 22587778
qscR	qscR	phzA2	phzA2	?	27242034*
qscR	qscR	rubA1	rubA1	?	27242034*
qscR	qscR	lasI	lasI	?	27242034*
qscR	qscR	phzF2	phzF2	?	27242034*
qscR	qscR	phzB1	phzB1	?	27242034*
qscR	qscR	phzB2	phzB2	?	27242034*
qscR	qscR	hcnA	hcnA	?	27242034*
qscR	qscR	hcnC	hcnC	?	27242034*
qscR	qscR	hcnB	hcnB	?	27242034*
qscR	qscR	phzD1	phzD1	?	27242034*
qscR	qscR	rhlI	rhlI	?	27242034*
qscR	qscR	phzA1	phzA1	?	27242034*
qscR	qscR	lasB	lasB	?	27242034*
qscR	qscR	PA4851_17455	PA1896	?	27242034*
qscR	qscR	PA4851_17460	PA1895	?	27242034*
qscR	qscR	PA4851_17465	PA1894	?	27242034*
qscR	qscR	PA4851_17470	PA1893	?	27242034*
qscR	qscR	PA4851_17475	PA1892	?	27242034*
qscR	qscR	PA4851_17480	PA1891	?	27242034*
qscR	qscR	phzG2	phzG2	?	27242034*
qscR	qscR	phzG1	phzG1	?	27242034*
qscR	qscR	phzC1	phzC1	?	27242034*
qscR	qscR	phzE1	phzE1	?	27242034*
RbsR	RbsR	rbsB	rbsB	-	27242034*
RbsR	RbsR	rbsA	rbsA	-	27242034*
RbsR	RbsR	rbsC	rbsC	-	27242034*
RbsR	RbsR	rbsR	rbsR	-	27242034*
RbsR	RbsR	rbsK	rbsK	-	27242034*
recA	recA	recA	recA	?	27242034*
rhlR	rhlR	acpP	acpP	+	27242034*, 18974177*, 22587778
rhlR	rhlR	PA4851_17590	PA1869	+	27242034*, 18974177*, 22587778
rhlR	rhlR	exoS	exoS	-	27242034*, 18974177*, 22587778
rhlR	rhlR	hcnA	hcnA	+	27242034*, 18974177*, 22587778
rhlR	rhlR	hcnB	hcnB	+	27242034*, 18974177*, 22587778
rhlR	rhlR	hcnC	hcnC	+	27242034*, 18974177*, 22587778
rhlR	rhlR	lecA	lecA	+	27242034*, 18974177*, 22587778
rhlR	rhlR	migA	migA	+	27242034*, 18974177*, 22587778

Regulatory gene	Ortholog of the regulatory gene	Target gene	Ortholog of the target gene	Mode of regulation	Reference (PubMed)
rhlR	rhlR	mvfR	mvfR	-	27242034*, 18974177*, 22587778
rhlR	rhlR	phzA1	phzA1	+	27242034*, 18974177*, 22587778
rhlR	rhlR	phzB1	phzB1	+	27242034*, 18974177*, 22587778
rhlR	rhlR	phzC1	phzC1	+	27242034*, 18974177*, 22587778
rhlR	rhlR	phzD1	phzD1	+	27242034*, 18974177*, 22587778
rhlR	rhlR	phzE1	phzE1	+	27242034*, 18974177*, 22587778
rhlR	rhlR	phzF1	phzF1	+	27242034*, 18974177*, 22587778
rhlR	rhlR	phzG1	phzG1	+	27242034*, 18974177*, 22587778
rhlR	rhlR	pqsA	pqsA	-	27242034*, 18974177*, 22587778
rhlR	rhlR	pqsB	pqsB	-	27242034*, 18974177*, 22587778
rhlR	rhlR	pqsC	pqsC	-	27242034*, 18974177*, 22587778
rhlR	rhlR	pqsD	pqsD	-	27242034*, 18974177*, 22587778
rhlR	rhlR	pqsE	pqsE	-	27242034*, 18974177*, 22587778
rhlR	rhlR	rhlA	rhlA	+	27242034*, 18974177*, 22587778
rhlR	rhlR	rhlB	rhlB	-	27242034*, 18974177*, 22587778
rhlR	rhlR	rhlG	rhlG	?	27242034*, 18974177*, 22587778
rhlR	rhlR	rhlI	rhlI	+	27242034*, 18974177*, 22587778
rhlR	rhlR	rhlR	rhlR	-	27242034*, 18974177*, 22587778
rhlR	rhlR	rpoS	rpoS	-	27242034*, 18974177*, 22587778
rhlR	rhlR	xcpW	xcpW	?	27242034*
rhlR	rhlR	xcpV	xcpV	?	27242034*
rhlR	rhlR	xcpU	xcpU	?	27242034*
rhlR	rhlR	xcpT	xcpT	?	27242034*
rhlR	rhlR	xcpS	xcpS	?	27242034*
rhlR	rhlR	xcpR	xcpR	?	27242034*
rhlR	rhlR	xcpQ	xcpQ	?	27242034*
rhlR	rhlR	phzF2	phzF2	?	27242034*
rhlR	rhlR	xcpZ	xcpZ	?	27242034*
rhlR	rhlR	xcpX	xcpX	?	27242034*
rhlR	rhlR	chiC	chiC	?	27242034*
rhlR	rhlR	PA4851_00920	PA0179	?	27242034*
rhlR	rhlR	lasA	lasA	?	27242034*
rhlR	rhlR	lasB	lasB	?	27242034*
rhlR	rhlR	lasI	lasI	?	27242034*
rhlR	rhlR	phzG2	phzG2	?	27242034*
rhlR	rhlR	xcpP	xcpP	?	27242034*
rocA1	rocA1	cupB1	cupB1	+	27242034*, 18974177*, 22587778
rocA1	rocA1	cupC1	cupC1	-	27242034*, 18974177*, 22587778
rocA1	rocA1	cupB4	cupB4	?	27242034*
rocA1	rocA1	rocR	rocR	?	27242034*
rocA1	rocA1	cupB2	cupB2	?	27242034*
rocA1	rocA1	rocS1	rocS1	?	27242034*
rocA1	rocA1	cupB6	cupB6	?	27242034*
rocA1	rocA1	cupB5	cupB5	?	27242034*
rocA1	rocA1	cupC3	cupC3	?	27242034*

Regulatory gene	Ortholog of the regulatory gene	Target gene	Ortholog of the target gene	Mode of regulation	Reference (PubMed)
rocA1	rocA1	cupB3	cupB3	?	27242034*
rocA1	rocA1	cupC2	cupC2	?	27242034*
rocA1	rocA1	rocA1	rocA1	?	27242034*
roxR	roxR	ccoN1	ccoN1	+	27242034*, 18974177*, 22587778
roxR	roxR	ccoN2	ccoN2	+	27242034*, 18974177*, 22587778
roxR	roxR	ccoO1	ccoO1	+	27242034*, 18974177*, 22587778
roxR	roxR	ccoO2	ccoO2	+	27242034*, 18974177*, 22587778
roxR	roxR	ccoP1	ccoP1	+	27242034*, 18974177*, 22587778
roxR	roxR	ccoP2	ccoP2	+	27242034*, 18974177*, 22587778
roxR	roxR	ccoQ1	ccoQ1	+	27242034*, 18974177*, 22587778
roxR	roxR	ccoQ2	ccoQ2	+	27242034*, 18974177*, 22587778
roxR	roxR	cioA	cioA	+	27242034*, 18974177*, 22587778
roxR	roxR	cioB	cioB	+	27242034*, 18974177*, 22587778
roxR	roxR	coxA	coxA	-	27242034*, 18974177*, 22587778
roxR	roxR	coxB	coxB	-	27242034*, 18974177*, 22587778
roxR	roxR	cyoA	cyoA	+	27242034*, 18974177*, 22587778
roxR	roxR	cyoB	cyoB	+	27242034*, 18974177*, 22587778
roxR	roxR	cyoC	cyoC	+	27242034*, 18974177*, 22587778
roxR	roxR	cyoD	cyoD	+	27242034*, 18974177*, 22587778
roxR	roxR	cyoE	cyoE	+	27242034*, 18974177*, 22587778
roxR	roxR	roxR	roxR	+	27242034*, 18974177*, 22587778
roxS	roxS	ccoN1	ccoN1	+	27242034*, 18974177*, 22587778
roxS	roxS	ccoN2	ccoN2	+	27242034*, 18974177*, 22587778
roxS	roxS	ccoO1	ccoO1	+	27242034*, 18974177*, 22587778
roxS	roxS	ccoO2	ccoO2	+	27242034*, 18974177*, 22587778
roxS	roxS	ccoP1	ccoP1	+	27242034*, 18974177*, 22587778
roxS	roxS	ccoP2	ccoP2	+	27242034*, 18974177*, 22587778
roxS	roxS	ccoQ1	ccoQ1	+	27242034*, 18974177*, 22587778
roxS	roxS	ccoQ2	ccoQ2	+	27242034*, 18974177*, 22587778
roxS	roxS	cioA	cioA	+	27242034*, 18974177*, 22587778
roxS	roxS	cioB	cioB	+	27242034*, 18974177*, 22587778
roxS	roxS	coxA	coxA	-	27242034*, 18974177*, 22587778
roxS	roxS	coxB	coxB	-	27242034*, 18974177*, 22587778
roxS	roxS	cyoA	cyoA	+	27242034*, 18974177*, 22587778
roxS	roxS	cyoB	cyoB	+	27242034*, 18974177*, 22587778
roxS	roxS	cyoC	cyoC	+	27242034*, 18974177*, 22587778
roxS	roxS	cyoD	cyoD	+	27242034*, 18974177*, 22587778
roxS	roxS	cyoE	cyoE	+	27242034*, 18974177*, 22587778
rpoD	rpoD	aotJ	aotJ	+	27242034*, 18974177*, 22587778
rpoD	rpoD	fleQ	fleQ	+	27242034*, 18974177*, 22587778
rpoD	rpoD	ptxR	ptxR	+	27242034*, 18974177*, 22587778
rpoD	rpoD	rpoS	rpoS	?	27242034*, 18974177*, 22587778
rpoD	rpoD	rpoD	rpoD	?	27242034*, 18974177*, 22587778
rpoN	rpoN	alg44	alg44	+	27242034*, 18974177*, 22587778
rpoN	rpoN	alg8	alg8	+	27242034*, 18974177*, 22587778

Regulatory gene	Ortholog of the regulatory gene	Target gene	Ortholog of the target gene	Mode of regulation	Reference (PubMed)
rpoN	rpoN	algA	algA	+	27242034*, 18974177*, 22587778
rpoN	rpoN	algB	algB	?	27242034*, 18974177*, 22587778
rpoN	rpoN	algD	algD	d	27242034*, 18974177*, 22587778
rpoN	rpoN	algE	algE	+	27242034*, 18974177*, 22587778
rpoN	rpoN	algF	algF	+	27242034*, 18974177*, 22587778
rpoN	rpoN	algG	algG	+	27242034*, 18974177*, 22587778
rpoN	rpoN	algI	algI	+	27242034*, 18974177*, 22587778
rpoN	rpoN	algJ	algJ	+	27242034*, 18974177*, 22587778
rpoN	rpoN	algK	algK	+	27242034*, 18974177*, 22587778
rpoN	rpoN	algL	algL	+	27242034*, 18974177*, 22587778
rpoN	rpoN	algX	algX	+	27242034*, 18974177*, 22587778
rpoN	rpoN	fhp	fhp	+	27242034*, 18974177*, 22587778
rpoN	rpoN	PA4851_11865	fhpR	+	27242034*, 18974177*, 22587778
rpoN	rpoN	fleN	fleN	+	27242034*, 18974177*, 22587778
rpoN	rpoN	fleR	fleR	+	27242034*, 18974177*, 22587778
rpoN	rpoN	fleS	fleS	+	27242034*, 18974177*, 22587778
rpoN	rpoN	flgB	flgB	+	27242034*, 18974177*, 22587778
rpoN	rpoN	flgC	flgC	+	27242034*, 18974177*, 22587778
rpoN	rpoN	flgD	flgD	+	27242034*, 18974177*, 22587778
rpoN	rpoN	flgE	flgE	+	27242034*, 18974177*, 22587778
rpoN	rpoN	flgF	flgF	+	27242034*, 18974177*, 22587778
rpoN	rpoN	flgG	flgG	+	27242034*, 18974177*, 22587778
rpoN	rpoN	flgH	flgH	+	27242034*, 18974177*, 22587778
rpoN	rpoN	flgI	flgI	+	27242034*, 18974177*, 22587778
rpoN	rpoN	flgJ	flgJ	+	27242034*, 18974177*, 22587778
rpoN	rpoN	flgK	flgK	+	27242034*, 18974177*, 22587778
rpoN	rpoN	flgL	flgL	+	27242034*, 18974177*, 22587778
rpoN	rpoN	flhA	flhA	+	27242034*, 18974177*, 22587778
rpoN	rpoN	flhB	flhB	+	27242034*, 18974177*, 22587778
rpoN	rpoN	flhF	flhF	+	27242034*, 18974177*, 22587778
rpoN	rpoN	fliD	fliD	+	27242034*, 18974177*, 22587778
rpoN	rpoN	fliE	fliE	+	27242034*, 18974177*, 22587778
rpoN	rpoN	fliF	fliF	+	27242034*, 18974177*, 22587778
rpoN	rpoN	fliG	fliG	+	27242034*, 18974177*, 22587778
rpoN	rpoN	PA4851_21790	fliH	+	27242034*, 18974177*, 22587778
rpoN	rpoN	fliI	fliI	+	27242034*, 18974177*, 22587778
rpoN	rpoN	fliJ	fliJ	+	27242034*, 18974177*, 22587778
rpoN	rpoN	PA4851_19770	PA1442	+	27242034*, 18974177*, 22587778
rpoN	rpoN	fliM	fliM	+	27242034*, 18974177*, 22587778
rpoN	rpoN	fliN	fliN	+	27242034*, 18974177*, 22587778
rpoN	rpoN	fliO	fliO	+	27242034*, 18974177*, 22587778
rpoN	rpoN	fliP	fliP	+	27242034*, 18974177*, 22587778
rpoN	rpoN	fliQ	fliQ	+	27242034*, 18974177*, 22587778
rpoN	rpoN	fliR	fliR	+	27242034*, 18974177*, 22587778
rpoN	rpoN	PA4851_21830	fliS	+	27242034*, 18974177*, 22587778

Regulatory gene	Ortholog of the regulatory gene	Target gene	Ortholog of the target gene	Mode of regulation	Reference (PubMed)
rpoN	rpoN	oprE	oprE	+	27242034*, 18974177*, 22587778
rpoN	rpoN	wbpI	orfK	+	27242034*, 18974177*, 22587778
rpoN	rpoN	wbpK	orfM	+	27242034*, 18974177*, 22587778
rpoN	rpoN	wbpL	orfN	+	27242034*, 18974177*, 22587778
rpoN	rpoN	PA4851_22010	phaG	+	27242034*, 18974177*, 22587778
rpoN	rpoN	proC	proC	+	27242034*, 18974177*, 22587778
rpoN	rpoN	rhlI	rhlI	-	27242034*, 18974177*, 22587778
rpoN	rpoN	rhlR	rhlR	+	27242034*, 18974177*, 22587778
rpoN	rpoN	PA4851_30400	sadB	?	27242034*, 18974177*, 22587778
rpoN	rpoN	glnA	glnA	?	27242034*, 18974177*, 22587778
rpoN	rpoN	gacA	gacA	?	27242034*
rpoN	rpoN	rhlB	rhlB	?	27242034*
rpoN	rpoN	rhlA	rhlA	?	27242034*
rpoN	rpoN	ureB	ureB	?	27242034*
rpoN	rpoN	ureA	ureA	?	27242034*
rpoN	rpoN	ureC	ureC	?	27242034*
rpoS	rpoS	azu	azu	+	27242034*, 18974177*, 22587778
rpoS	rpoS	bphO	bphO	+	27242034*, 18974177*, 22587778
rpoS	rpoS	bphP	bphP	+	27242034*, 18974177*, 22587778
rpoS	rpoS	cioA	cioA	+	27242034*, 18974177*, 22587778
rpoS	rpoS	cioB	cioB	+	27242034*, 18974177*, 22587778
rpoS	rpoS	coIII	coIII	+	27242034*, 18974177*, 22587778
rpoS	rpoS	coxA	coxA	+	27242034*, 18974177*, 22587778
rpoS	rpoS	coxB	coxB	+	27242034*, 18974177*, 22587778
rpoS	rpoS	exsA	exsA	+	27242034*, 18974177*, 22587778
rpoS	rpoS	exsB	exsB	+	27242034*, 18974177*, 22587778
rpoS	rpoS	exsC	exsC	+	27242034*, 18974177*, 22587778
rpoS	rpoS	exsE	exsE	+	27242034*, 18974177*, 22587778
rpoS	rpoS	rhlI	rhlI	-	27242034*, 18974177*, 22587778
rpoS	rpoS	hcnA	hcnA	?	27242034*, 18974177*, 22587778
rpoS	rpoS	phzC1	phzC1	?	27242034*, 18974177*, 22587778
rpoS	rpoS	phzF1	phzF1	?	27242034*, 18974177*, 22587778
rpoS	rpoS	lasB	lasB	?	27242034*, 18974177*, 22587778
rpoS	rpoS	lecA	lecA	?	27242034*
rpoS	rpoS	aer2	aer2	?	27242034*
rpoS	rpoS	phzF2	phzF2	?	27242034*
rpoS	rpoS	phzD1	phzD1	?	27242034*
rpoS	rpoS	phzB1	phzB1	?	27242034*
rpoS	rpoS	phzG1	phzG1	?	27242034*
rpoS	rpoS	hcnC	hcnC	?	27242034*
rpoS	rpoS	hcnB	hcnB	?	27242034*
rpoS	rpoS	PA4851_17285	PA1930	?	27242034*
rpoS	rpoS	cttP	cttP	?	27242034*
rpoS	rpoS	PA4851_00920	PA0179	?	27242034*
rpoS	rpoS	PA4851_00915	PA0178	?	27242034*

Regulatory gene	Ortholog of the regulatory gene	Target gene	Ortholog of the target gene	Mode of regulation	Reference (PubMed)
rpoS	rpoS	PA4851_00910	PA0177	?	27242034*
rpoS	rpoS	phzA1	phzA1	?	27242034*
rpoS	rpoS	phzG2	phzG2	?	27242034*
rpoS	rpoS	lasA	lasA	?	27242034*
rpoS	rpoS	PA4851_13455	PA2573	?	27242034*
rpoS	rpoS	phzE1	phzE1	?	27242034*
rsaL	rsaL	phzA1	phzA1	-	27242034*, 18974177*, 22587778
rsaL	rsaL	lasI	lasI	-	27242034*, 18974177*, 22587778
rsaL	rsaL	lasB	lasB	?	27242034*, 18974177*, 22587778
rsaL	rsaL	rsaL	rsaL	-	27242034*, 18974177*, 22587778
rsaL	rsaL	phzM	phzM	-	27242034*, 18974177*, 22587778
rsaL	rsaL	hcnA	hcnA	-	27242034*, 18974177*, 22587778
rsaL	rsaL	phzF1	phzF1	?	27242034*
rsaL	rsaL	phzF2	phzF2	?	27242034*
rsaL	rsaL	phzB1	phzB1	?	27242034*
rsaL	rsaL	phzC1	phzC1	?	27242034*
rsaL	rsaL	phzE1	phzE1	?	27242034*
rsaL	rsaL	phzD1	phzD1	?	27242034*
rsaL	rsaL	phzG1	phzG1	?	27242034*
rsaL	rsaL	lasI	lasI	?	27242034*
rsaL	rsaL	phzG2	phzG2	?	27242034*
rsaL	rsaL	hcnC	hcnC	?	27242034*
rsaL	rsaL	hcnB	hcnB	?	27242034*
soxR	soxR	PA4851_06240	PA3718	+	27242034*, 18974177*, 22587778
soxR	soxR	mexG	mexG	+	27242034*, 18974177*, 22587778
soxR	soxR	mexH	mexH	+	27242034*, 18974177*, 22587778
soxR	soxR	mexI	mexI	+	27242034*, 18974177*, 22587778
soxR	soxR	opmD	opmD	+	27242034*, 18974177*, 22587778
soxR	soxR	PA4851_15270	PA2274	+	27242034*, 18974177*, 22587778
soxR	soxR	soxR	soxR	+	27242034*, 18974177*, 22587778
toxR	toxR	oprL	oprL	+	27242034*, 18974177*, 22587778
toxR	toxR	PA4851_05610	PA3842	+	27242034*, 18974177*, 22587778
toxR	toxR	motD	motD	+	27242034*, 18974177*, 22587778
toxR	toxR	tolA	tolA	+	27242034*, 18974177*, 22587778
toxR	toxR	tolB	tolB	+	27242034*, 18974177*, 22587778
toxR	toxR	tolQ	tolQ	+	27242034*, 18974177*, 22587778
toxR	toxR	tolR	tolR	+	27242034*, 18974177*, 22587778
toxR	toxR	toxA	toxA	-	27242034*, 18974177*, 22587778
tpbA	tpbA	PA4851_04145	PA4139	-	27242034*, 18974177*, 22587778
tpbA	tpbA	PA4851_26630	PA4624	-	27242034*, 18974177*, 22587778
tpbA	tpbA	PA4851_26635	PA4625	-	27242034*, 18974177*, 22587778
tpbA	tpbA	pelA	pelA	-	27242034*, 18974177*, 22587778
tpbA	tpbA	pelB	pelB	-	27242034*, 18974177*, 22587778
tpbA	tpbA	pelC	pelC	-	27242034*, 18974177*, 22587778
tpbA	tpbA	pelD	pelD	-	27242034*, 18974177*, 22587778

Regulatory gene	Ortholog of the regulatory gene	Target gene	Ortholog of the target gene	Mode of regulation	Reference (PubMed)
tpbA	tpbA	pelE	pelE	-	27242034*, 18974177*, 22587778
tpbA	tpbA	pelF	pelF	-	27242034*, 18974177*, 22587778
tpbA	tpbA	pelG	pelG	-	27242034*, 18974177*, 22587778
tpbA	tpbA	tpbA	tpbA	-	27242034*, 18974177*, 22587778
tpbA	tpbA	tpbB	tpbB	-	27242034*, 18974177*, 22587778
trpI	trpI	trpA	trpA	+	27242034*
trpI	trpI	trpB	trpB	+	27242034*
trpI	trpI	trpI	trpI	-	27242034*
vfr	vfr	cbpA	cbpA	+	27242034*, 18974177*, 22587778
vfr	vfr	fleQ	fleQ	-	27242034*, 18974177*, 22587778
vfr	vfr	lasR	lasR	+	27242034*, 18974177*, 22587778
vfr	vfr	ptxR	ptxR	+	27242034*, 18974177*, 22587778
vfr	vfr	toxR	toxR	+	27242034*, 18974177*, 22587778
vfr	vfr	rhlR	rhlR	d	27242034*, 18974177*, 22587778
vfr	vfr	toxA	toxA	+	27242034*, 18974177*, 22587778
vfr	vfr	vfr	vfr	+	27242034*, 18974177*, 22587778
vfr	vfr	pilP	pilP	?	27242034*
vfr	vfr	plcH	plcH	?	27242034*
vfr	vfr	PA4851_03305	PA0653	?	27242034*
vfr	vfr	plcN	plcN	?	27242034*
vfr	vfr	plcR	plcR	?	27242034*
vfr	vfr	pilM	pilM	?	27242034*
vfr	vfr	pilO	pilO	?	27242034*
vfr	vfr	pilN	pilN	?	27242034*
vfr	vfr	alg8	alg8	?	27242034*
vfr	vfr	algZ	algZ	?	27242034*
vfr	vfr	alg44	alg44	?	27242034*
vfr	vfr	algJ	algJ	?	27242034*
vfr	vfr	algK	algK	?	27242034*
vfr	vfr	algI	algI	?	27242034*
vfr	vfr	algL	algL	?	27242034*
vfr	vfr	lasI	lasI	?	27242034*
vfr	vfr	algA	algA	?	27242034*
vfr	vfr	algF	algF	?	27242034*
vfr	vfr	algG	algG	?	27242034*
vfr	vfr	algD	algD	?	27242034*
vfr	vfr	algE	algE	?	27242034*
vfr	vfr	exoT	exoT	?	27242034*
vfr	vfr	argH	argH	?	27242034*
vfr	vfr	pbpG	pbpG	?	27242034*
vfr	vfr	algX	algX	?	27242034*
vqsM	vqsM	pprB	pprB	+	27242034*, 18974177*, 22587778
vqsM	vqsM	rpoS	rpoS	+	27242034*, 18974177*, 22587778
vqsM	vqsM	PA4851_12250	PA2591	+	27242034*, 18974177*, 22587778
vreI	vreI	PA4851_24145	PA0691	+	27242034*
vreI	vreI	PA4851_14345	tpsB	+	27242034*

**Information retrieved from databases.*

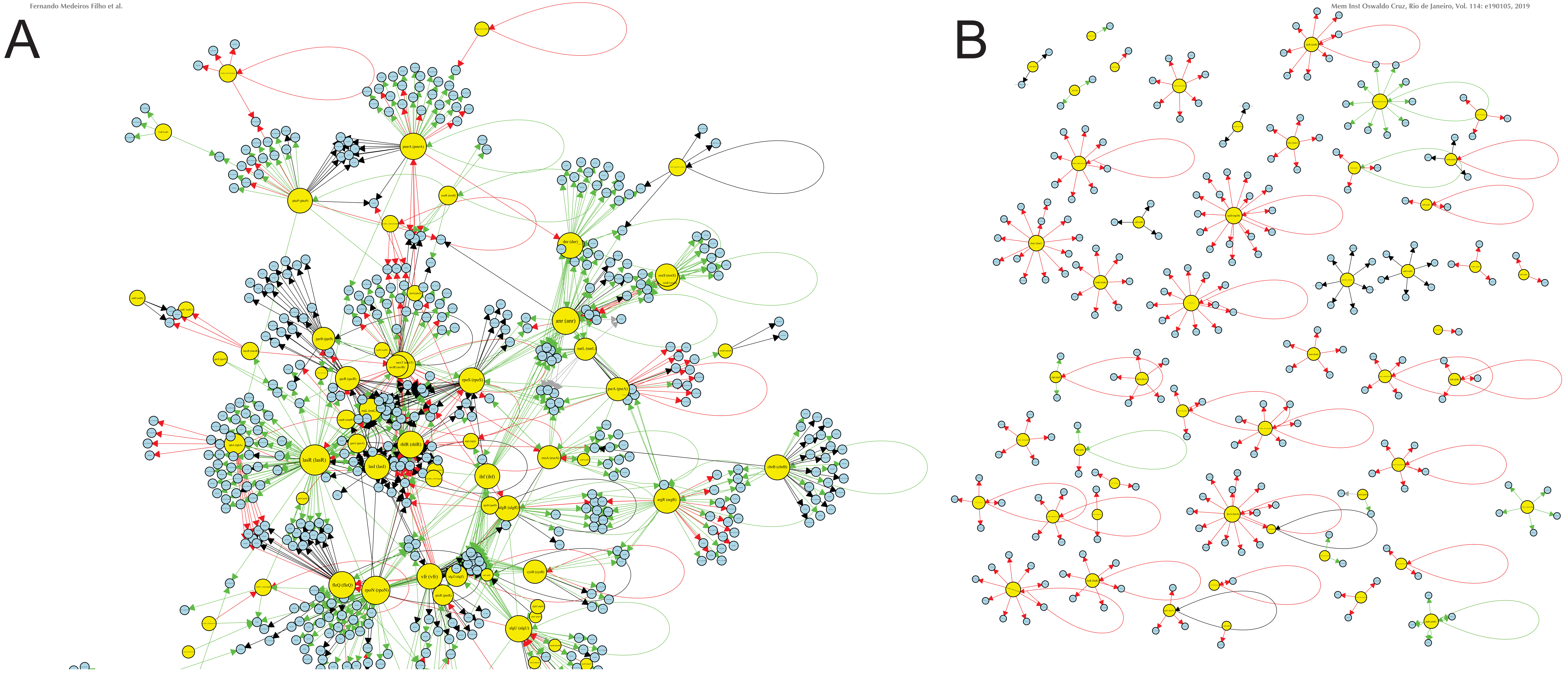

Fig. 2. Visualisation of the *Pseudomonas aeruginosa* CCBH4851 gene regulatory network (GRN). Yellow circles indicate regulatory genes, light blue circles indicate target genes (TGs), black lines indicate an unknown mode of regulation, green lines indicate activation, red lines indicate repression and grey lines indicate a dual mode of regulation. A: the GRN large highly connected network component; B: all regulatory and TGs that have no connections with the component depicted in A; C-E: clusters of lower connectivity compared to the component depicted in A.

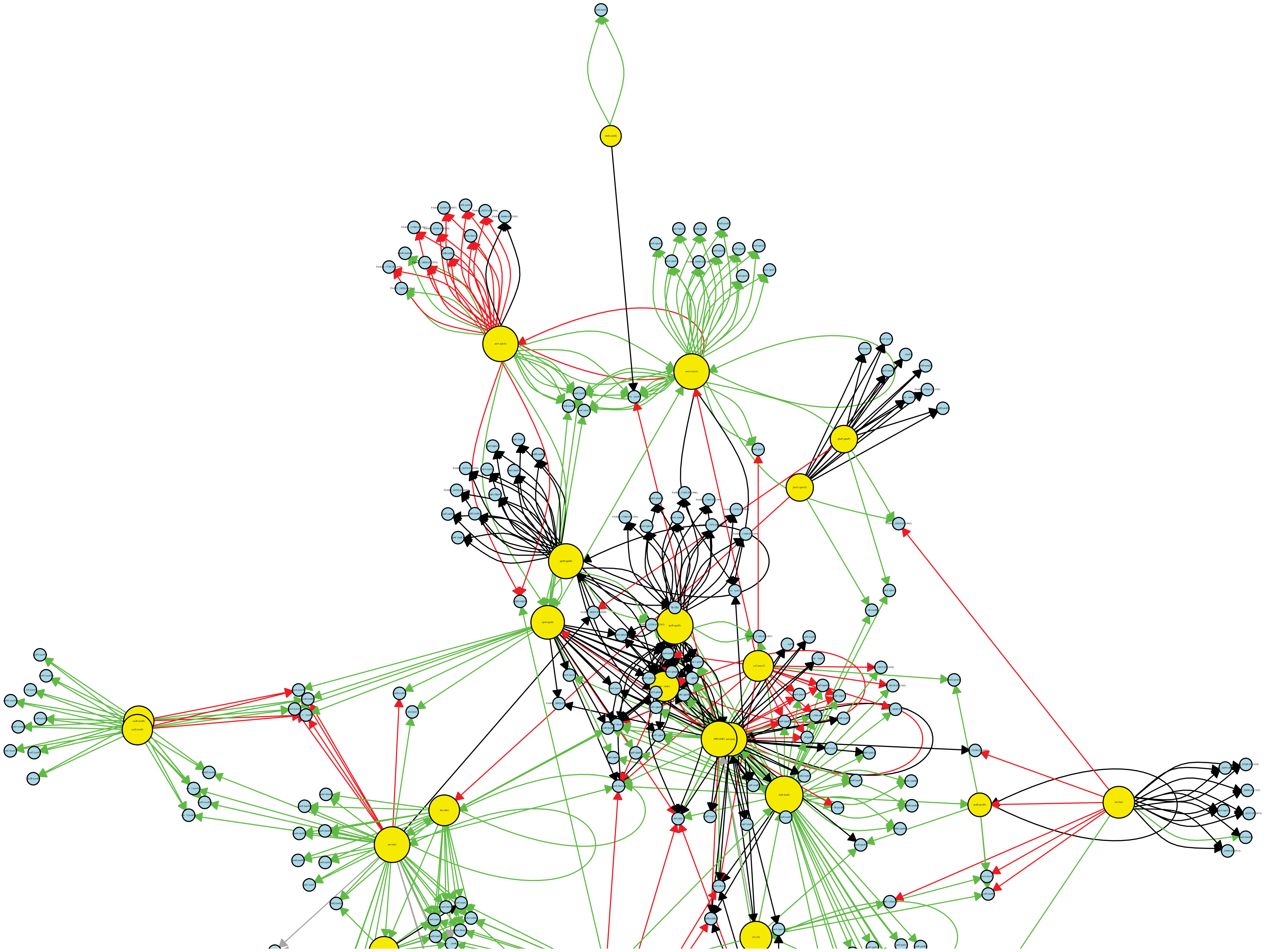

Fig. 4. Connectivity relationships among the 30 most influential hubs of the *Pseudomonas aeruginosa* CCBH4851 gene regulatory network. Yellow circles indicate regulatory genes considered hubs, light blue circles indicate target genes, black lines indicate an unknown mode of regulation, green lines indicate activation, red lines indicate repression and grey lines indicate a dual mode of regulation.

RBH algorithm

```
Usage = """RBH BLASTOUTPUT1 BLASTOUTPUT2 RBH-list-outfile """

import sys, re

if len(sys.argv) < 3:
      print(Usage)

debug = 9

infl1 = sys.argv[1]
infl2 = sys.argv[2]
outfile = sys.argv[3]

#parse first BLAST results
FL1 = open(infl1, 'r')
D1 = {} #dictionary for BLAST file ONE
for Line in FL1:
      if ( Line[0] != '#' ):
            Line.strip()
            Elements = re.split('\t', Line)
            queryId = Elements[0]
            subjectId = Elements[1]
            if ( not ( queryId in D1.keys() ) ):
                  D1[queryId] = subjectId  #pick the first hit

if (debug): D1.keys()

#parse second BLAST results
FL2 = open(infl2, 'r')
D2 = {}
for Line in FL2:
      if ( Line[0] != '#' ):
            Line.strip()
            Elements = re.split('\t', Line)
            queryId = Elements[0]
            subjectId = Elements[1]
            if ( not ( queryId in D2.keys() ) ):
                  D2[queryId] = subjectId  #pick the first hit

if (debug): D2.keys()

#Now, pick the share pairs

SharedPairs={}
for id1 in D1.keys():
      value1 = D1[id1]
      if ( value1 in D2.keys() ):
            if ( id1 == D2[value1] ) : #a shared best reciprocal
pair
                  SharedPairs[value1] = id1

if (debug): SharedPairs

#outfl = open("_out.csv", "w")
outfl = open( outfile, 'w')

for k1 in SharedPairs.keys():
      line = k1 + '\t' + SharedPairs[k1] + '\n'
```

```
        outfl.write(line)

    outfl.close()

    print("Done. RBH from", sys.argv[1], "and", sys.argv[2], "are in",
    sys.argv[3])
```

Topology Rcode

```
# packages 1
library(dplyr)
library(tibble)
library(readr)
# packages 2
library(igraph)
library(scales)


dados <-
  read_csv2("GRN.csv")

dados


c1 <-
  dados$`Regulator (TF or sigma)` %>%
  strsplit(" ") %>%
  unlist()

c1.TF <- c1[gtools::odd(1:length(c1))]
c1.ortologo.TF <- c1[gtools::even(1:length(c1))]


dados$`Regulator (TF)` <- c1.TF
dados$`orthologs of TF` <- c1.ortologo.TF


nrow(dados)  ==
  sum(paste(dados$`Regulator (TF)`,dados$`orthologs of TF`) ==
dados$`Regulator (TF or sigma)`)


dados <-
  dados %>%
  select(`Regulator (TF)`,
         `Target gene`,
         `mode of regulation`,
         `orthologs of TF`,
         `Ortholog of the target gene`
  )


dados$`orthologs of TF` <- gsub("\\(|\\)", "",
                                dados$`orthologs of TF`)



rm(c1, c1.ortologo.TF,c1.TF)


auxTF <- dados[,c(1,4)] %>% setNames(c("gene_CCBH4851",
"orthologs"))
auxTarget <- dados[,c(2,5)] %>% setNames(c("gene_CCBH4851",
"orthologs"))

vert <-
  dplyr::union(auxTF,auxTarget) %>%
```

```
  filter(!is.na(gene_CCBH4851) )

vert$rotulo <- paste0(vert$gene_CCBH4851,
                      " (",
                      vert$orthologs,
                      ")")


arestas <-
  dados[,c(1,2,3)] %>%
  filter(!is.na(`Target gene`))


Rede <- graph_from_data_frame(d = arestas,
                              directed = TRUE,
                              vertices = vert
)



V(Rede)$color <- ifelse(V(Rede)$name %in% auxTF$gene_CCBH4851,
                        "yellow", "lightblue")


codificacao <- '"+" = "green" ; "-" = "red" ; "?" = "white" ; "d" =
"darkgrey"'
E(Rede)$color <- car::Recode(E(Rede)$`mode of regulation`,
                             codificacao)


V(Rede)$size <- 2+ log(1+degree(Rede, mode = "out"))

V(Rede)$name <- V(Rede)$rotulo


set.seed(1234)
#
plot(Rede,
     layout=layout_nicely,
     vertex.label.dist=0,
     vertex.label.color='black',
     vertex.label.font=0.05,
     vertex.label.cex=0.7,
     edge.arrow.size=0.10,edge.arrow.width=1.8,
     edge.width=0.6
)
title(sub="RRG CCBH4851", cex.sub = 0.75, font.sub = 3, col.sub =
"black")


set.seed(2397)

plot(Rede,
     layout=layout_with_fr,
     vertex.label.dist=0,
     vertex.label.color='black',
     vertex.label.font=1,
     vertex.label.cex=1,
```

```
     edge.arrow.size=0.025,edge.arrow.width=0.7,
     edge.width=0.06
)
title(sub="RRG CCBH4851", cex.sub = 0.75, font.sub = 3, col.sub =
"black")


V(Rede)$name <- vert$gene_CCBH4851

nrow(vert)

nrow(arestas)

sum(V(Rede)$color == "yellow")


sum(V(Rede)$color == "lightblue")



scientific(graph.density(Rede, loops=TRUE))

p.kin <- degree_distribution(Rede, mode="in")
p.kin.na <- ifelse(p.kin == 0,NA,p.kin)
min.kin <- min(degree(Rede, mode="in"))
max.kin <-  max(degree(Rede, mode="in"))
plot(min.kin:max.kin, p.kin.na,
     xlab= "k-in (grau input)", ylab= "P(k-in)", type="h")
title(sub="Figura 2: DistribuiÃ§Ã£o de Grau k-in",
      cex.sub = 0.75, font.sub = 3, col.sub = "black")


x.in <- log10(min.kin:max.kin)
y.in <- log10(p.kin.na)
data.in <-
  data.frame(X=x.in,Y=y.in) %>%
  filter(!is.na(X) & !is.na(Y) & X != -Inf)
ajuste.in <- lm(Y~X,data=data.in)
log.A.in <- ajuste.in$coefficients[1]
A.in <- 10^(log.A.in)
gama.in <- -ajuste.in$coefficients[2]
#
plot(x.in, y.in,
     xlab= "log(k-in)", ylab= "log P(k-in)")
plotrix::ablineclip(log.A.in, -gama.in, x1= 0,x2=log10(max.kin))


p.kout <- degree_distribution(Rede, mode="out")
p.kout.na <- ifelse(p.kout == 0,NA,p.kout)
min.kout <- min(degree(Rede, mode="out"))
max.kout <- max(degree(Rede, mode="out"))
#
plot(min.kout:max.kout, p.kout.na,
     xlab= "k-out (grau output)", ylab= "P(k-out)", type="h")
title(sub="Figura 3: DistribuiÃ§Ã£o de Grau k-out",
      cex.sub = 0.75, font.sub = 3, col.sub = "black")

plot(min.kout:max.kout, p.kout.na,
```

```
     xlab= "k-out (grau output)", ylab= "P(k-out)",
type="h",log="y")
title(sub="Figura 4: DistribuiÃ§Ã£o de Grau k-out",
      cex.sub = 0.75, font.sub = 3, col.sub = "black")


x.out <- log10(min.kout:max.kout)
y.out <- log10(p.kout.na)
data.out <-
  data.frame(X=x.out,Y=y.out) %>%
  filter(!is.na(X) & !is.na(Y) & X != -Inf)
ajuste.out <- lm(Y~X,data=data.out)
log.A.out <- ajuste.out$coefficients[1]
A.out <- 10^(log.A.out)
gama.out <- -ajuste.out$coefficients[2]
#
plot(x.out, y.out,
     xlab= "log(k-out)", ylab= "log P(k-out)")
title(sub="Figura 5: DistribuiÃ§Ã£o de Grau k-out log-log",
      cex.sub = 0.75, font.sub = 3, col.sub = "black")
plotrix::ablineclip(log.A.out, -gama.out, x1= 0,x2=log10(max.kout))


CoeffCluster.global <-
scientific(transitivity(Rede,type="globalundirected"))

CoeffCluster.medio <- scientific(transitivity(Rede,type="average"))

CoeffCluster.i <- transitivity(Rede,type="localundirected",
                               isolates = "zero")

hist(CoeffCluster.i,
     xlab= "coeficiente de clusterizaÃ§Ã£o local", ylab=
"frequÃªncia", main=NULL)
title(sub="Figura 6: DistribuiÃ§Ã£o Total de Coef.
ClusterizaÃ§Ã£o",
      cex.sub = 0.75, font.sub = 3, col.sub = "black")

propCzero <-table(CoeffCluster.i)[1]/nrow(vert)


propChum <-
table(CoeffCluster.i)[nrow(table(CoeffCluster.i))]/nrow(vert)



hist(ifelse(CoeffCluster.i ==0 | CoeffCluster.i ==1, NA,
CoeffCluster.i),
     xlab= "coeficiente de clusterizaÃ§Ã£o local", ylab=
"frequÃªncia", main=NULL)
title(sub="Figura 2: DistribuiÃ§Ã£o Parcial de Coef.
ClusterizaÃ§Ã£o",
      cex.sub = 0.75, font.sub = 3, col.sub = "black")


k.i <- degree(Rede,mode="all")
```

```
C.k.i <- CoeffCluster.i
#
plot(k.i, C.k.i,
     xlab="k (grau total)", ylab= "C(k) (coef. cluster. por grau
k)")
title(sub="Figura 7: Coef. ClusterizaÃ§Ã£o em funÃ§Ã£o do grau k
dos vÃ©rtices ",
      cex.sub = 0.75, font.sub = 3, col.sub = "black")


C.k.i.filtrado <- transitivity(Rede,type="localundirected",
                               isolates = "NaN")

plot(k.i, C.k.i.filtrado,
     xlab="k (grau total)", ylab= "C(k) (coef. cluster. por grau
k)")
title(sub="Figura 7: Coef. ClusterizaÃ§Ã£o em funÃ§Ã£o do grau k
dos vÃ©rtices ",
      cex.sub = 0.75, font.sub = 3, col.sub = "black")


count_components(Rede)


components(Rede)$csize

plot(components(Rede)$csize)


reguladores.por.grupo <- c()
for(i in 1:count_components(Rede)){
  grupo <-
names(components(Rede)$membership)[components(Rede)$membership ==
i]
  reguladores.por.grupo<-
    c(reguladores.por.grupo,
      sum(grupo %in% auxTF$gene_CCBH4851))
}


propor.reguladores.por.grupo <-
reguladores.por.grupo/components(Rede)$csize


probab.n.reguladores.por.grupo <-
  table(reguladores.por.grupo)/count_components(Rede)

plot(probab.n.reguladores.por.grupo,
     log= "xy",
     ylim=c(0.01,1))


tab.modoRegula <-
  table(arestas$`mode of regulation`)
tab.modoRegula <- tab.modoRegula[order(tab.modoRegula)]

auto_regul <-
  filter(arestas, `Regulator (TF)` == `Target gene`)
```

```
#
tab.modo.Auto_regul <-
  table(auto_regul$`mode of regulation`)
tab.modo.Auto_regul <-
tab.modo.Auto_regul[order(tab.modo.Auto_regul)]

diameter(Rede,directed = TRUE,unconnected = TRUE)
diameter(Rede,directed = FALSE,unconnected = TRUE)

mean_distance(Rede, directed = TRUE, unconnected = TRUE)
mean_distance(Rede, directed = FALSE, unconnected = TRUE)


triad_census(Rede)

triad_census(Rede)[9]

Rede2 <- graph_from_data_frame(d = filter(arestas,
                                          `mode of regulation` ==
"+" |
                                            `mode of regulation` ==
"-"),
                               directed = TRUE, vertices = vert )

triad_census(Rede2)

triad_census(Rede2)[9]


Rede3 <- graph_from_data_frame(d = filter(arestas,
                                          `mode of regulation` ==
"+" ),
                               directed = TRUE, vertices = vert
)


triad_census(Rede3)

triad_census(Rede3)[9]


Rede4 <- graph_from_data_frame(d = filter(arestas,
                                          `mode of regulation` ==
"-" ),
                               directed = TRUE, vertices = vert
)

triad_census(Rede4)

triad_census(Rede4)[9]


V(Rede)$name <- V(Rede)$rotulo



hubs.em.ordem.dec <-
(hub_score(Rede)$vector)[order((hub_score(Rede)$vector),
```

```
                                                 decreasing =
TRUE)]

authority.em.ordem.dec <-

authority_score(Rede)$vector[order((authority_score(Rede)$vector),
                                    decreasing = TRUE)]


hubs.em.ordem.dec[1:10]

authority.em.ordem.dec[1:10]

k.hubs.em.ordem.dec <- degree(Rede, mode="out")[order(degree(Rede,
mode="out"),
                                                    decreasing =
TRUE)]
k.hubs.em.ordem.dec[1:33]
```
